# Achieving very bright mechanoluminescence from purely organic luminophores with aggregation-induced emission by crystal design†
†Electronic supplementary information (ESI) available: Details of the synthesis; structural information for the compounds (NMR, elemental analysis and mass spectra), Fig. S1–S31 and videos S1–S5. CCDC 1468361–1468365. For ESI and crystallographic data in CIF or other electronic format see DOI: 10.1039/c6sc01325b


**DOI:** 10.1039/c6sc01325b

**Published:** 2016-04-26

**Authors:** Bingjia Xu, Wenlang Li, Jiajun He, Sikai Wu, Qiangzhong Zhu, Zhiyong Yang, Yuan-Chun Wu, Yi Zhang, Chongjun Jin, Po-Yen Lu, Zhenguo Chi, Siwei Liu, Jiarui Xu, Martin R. Bryce

**Affiliations:** a PCFM Lab , GD HPPC Lab , Guangdong Engineering Technology Research Center for High-performance Organic and Polymer Photoelectric Functional Films , State Key Laboratory of Optoelectronic Material and Technologies , School of Chemistry and Chemical Engineering , Sun Yat-sen University , Guangzhou 510275 , China . Email: yangzhy29@mail.sysu.edu.cn ; Email: ceszy@mail.sysu.edu.cn ; Email: chizhg@mail.sysu.edu.cn ; Fax: +86 20 84112222 ; Tel: +86 20 84112712; b State Key Laboratory of Optoelectronic Material and Technologies , School of Physics and Engineering , Sun Yat-sen University , Guangzhou 510275 , China; c Shenzhen China Star Optoelectronics Technology Co., Ltd , Shenzhen 518107 , China; d Department of Chemistry , Durham University , DH1 3LE , UK

## Abstract

Although bright organic mechanoluminescence (ML) has been observed for a few luminophores with aggregation-induced emission (AIE), details of the positive effect of AIE on ML performance remain unclear and a feasible design principle for AIE-ML compounds has not yet been presented. Herein, an effective strategy for the molecular design of efficient AIE-ML materials is demonstrated, based on tetraphenylethene (TPE) building blocks with formyl substituents, which yield non-centrosymmetric crystal structures with prominent piezoelectric properties for molecular excitation combined with AIE features for intense emission. Following this approach, three AIE-active compounds have been developed and are found to show unique ML characteristics. Furthermore, the results of single crystal X-ray analysis and density functional theory (DFT) calculations suggest that the ML performance would probably be further enhanced by creating molecules with larger dipolar moments and enhanced AIE properties.

## Abstract 2

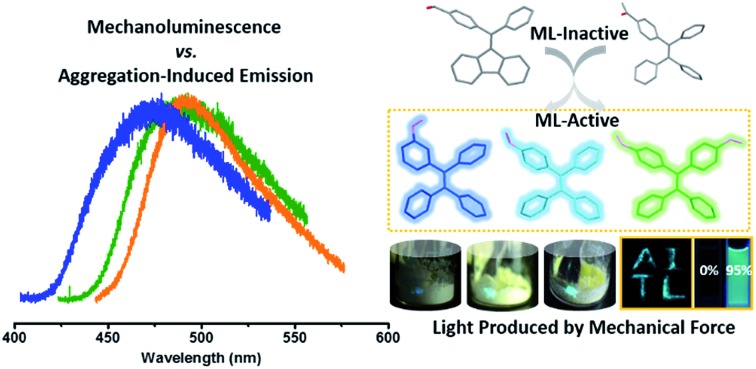
An effective strategy for the molecular design of AIE-ML materials is demonstrated based on tetraphenylethene with formyl substituents.

## Introduction

Mechanoluminescence (ML), which is a type of light emission induced by mechanical force on crystals, has attracted increasing interest due to its special photophysical process and potential applications in displays, lighting, bioimaging and stress sensing.^1^ The first record of this vivid phenomenon dates back to 1605, when Francis Bacon noted that lumps of sugar being scraped at night would afford a sparkling light.^2^ Since then, certain inorganic compounds and organometallic complexes, such as manganese-doped zinc sulfide, Eu and Dy co-doped strontium aluminates, europium tetrakis(dibenzoylmethide) triethylammonium, ditriphenyl phosphine oxide manganese bromide, and bis(methyl triphenylphosphonium)dibromodichloromanganate, have been reported to show remarkable ML at room temperature.^3^ However, by contrast, purely organic dyes which are capable of generating intense ML are extremely scarce, and heretofore, only a few examples have been reported to display observable ML even at cryogenic temperatures or in the dark.3c,^4^ Due to their poor performance, metal-free organic compounds fail to enter discussions on practical ML applications. One of the most serious obstacles to the development of efficient purely organic ML materials is the notorious aggregation-caused quenching (ACQ) effect, which originates from strong π–π stacking interactions that greatly impede efficient emission from organic molecules.^5^ Consequently, bright ML is difficult to achieve in purely organic luminophores and, generally, has been regarded as a solely inorganic or organometallic property for the past four hundred years.1d,^6^

Recently, three metal-free organic compounds, namely 10-(4-(4-(9*H*-carbazol-9-yl)phenylsulfonyl)phenyl)-10*H*-phenothiazine (SFPC, Fig. S1a†), 5-(4-(1,2,2-triphenylvinyl)phenyl)thiophene-2-carbaldehyde (P_4_TA, Fig. S1b†) and 1,1,2,2-tetrakis(4-methoxyphenyl)ethane (TMPE, Fig. S1c†) with ML properties have been prepared by our group and by Li *et al.*^7^ The unique aggregation-induced emission (AIE) character of SFPC, P_4_TA and TMPE surmounted the intrinsic ACQ effect of conventional organic dyes, thus resulting in high fluorescence quantum yields (*Φ*_F,s_) in the solid state and intense ML even in daylight at room temperature. These three publications also provide a new direction for the development of efficient organic ML materials by combining prominent piezoelectric properties for molecular excitation and AIE phenomena for emission. AIE luminophores (*e.g.* tetraphenylethene derivatives) commonly adopt twisted conformations, which serve to effectively avoid π–π stacking and thereby lead to dramatic emission enhancement in the solid state with respect to in dilute solution.^8^ Accordingly, compounds with AIE properties should be ideal candidates for the generation of strong ML. However, the molecular structures of current AIE-ML compounds are varied, therefore, the ML activity of an AIE compound is unpredictable. Furthermore, for this novel type of luminophores, details of the positive effect of AIE on ML performance remain unclear. Notably, although thousands of AIE luminophores with various functions have been documented, to date, none of them have been demonstrated to show ML activity except for the SFPC, P_4_TA and TMPE compounds noted above; this is probably owing to the weak piezoelectric effect of the corresponding crystals.8c,^9^ As previously reported, organic crystals constructed with dipolar molecules and employing non-centrosymmetric molecular arrangements should favor piezoelectric properties which is closely pertinent to the ML activity.2b,3b,4b,^10^ Nevertheless, a comprehensive understanding of the molecular structure requirements for obtaining non-centrosymmetric organic crystals, especially those with AIE features, is less well demonstrated. Such a deficiency in understanding means that a coherent design principle for ML materials with AIE activity has not been reported so far.

In this article, we have identified that employing a tetraphenylethene (TPE) moiety functionalized with formyl groups as building blocks is beneficial for the construction of non-centrosymmetric organic crystals with AIE properties and yielding high brightness ML. Herein, formyl groups are chosen as functional substituents on TPE, because they are typical electron withdrawing units with small sizes, which allows the resulting molecules to achieve large intramolecular dipole moments and to form strong intermolecular hydrogen bonds to facilitate crystallization with close packing. Following this strategy, another two new organic compounds have been developed and are found to show AIE-ML properties as predicted. Meanwhile, the relationship between AIE performance and ML enhancement is also demonstrated in this study. This work represents the first effective strategy for the molecular design of efficient AIE-ML materials.

## Results and discussion

**Fig. 1:**
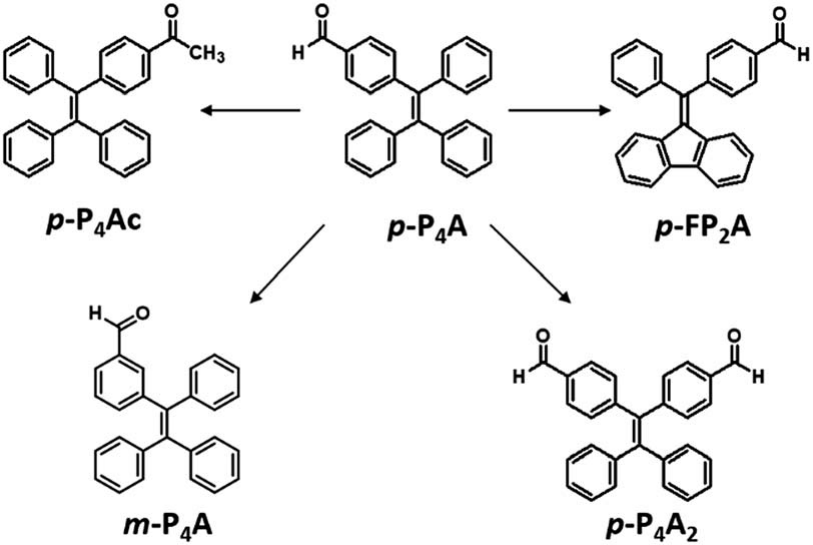
Molecular structures of the target compounds.

**Fig. 2:**
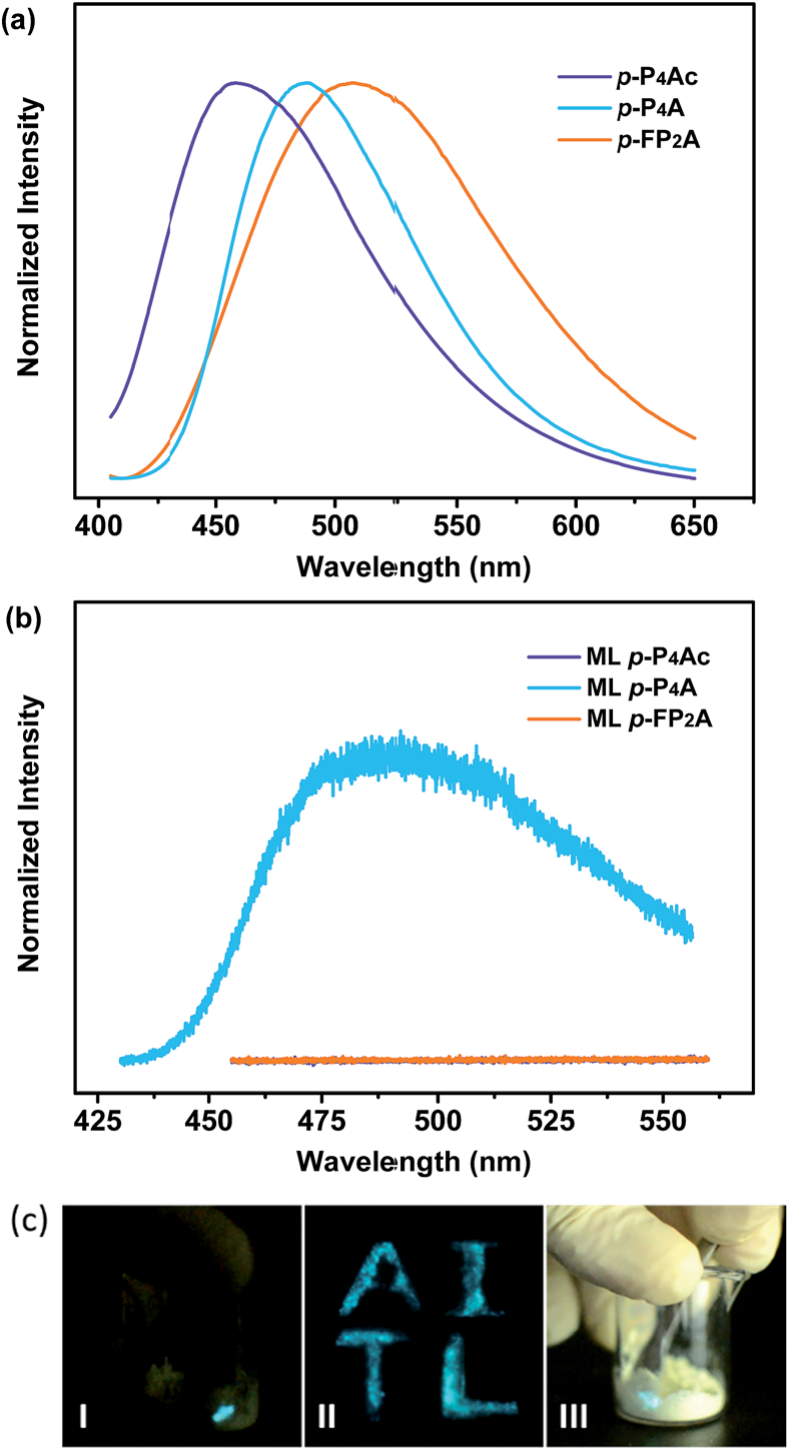
PL spectra (a) and ML spectra (b) of *p*-P_4_Ac, *p*-P_4_A and *p*-FP_2_A; (c) ML images of *p*-P_4_A: (I) performed in dark, (II) capital letters ‘AITL’ displayed using ML in the dark; (III) performed under daylight. The PL spectra were recorded under excitation of 365 nm UV light.

**Fig. 3:**
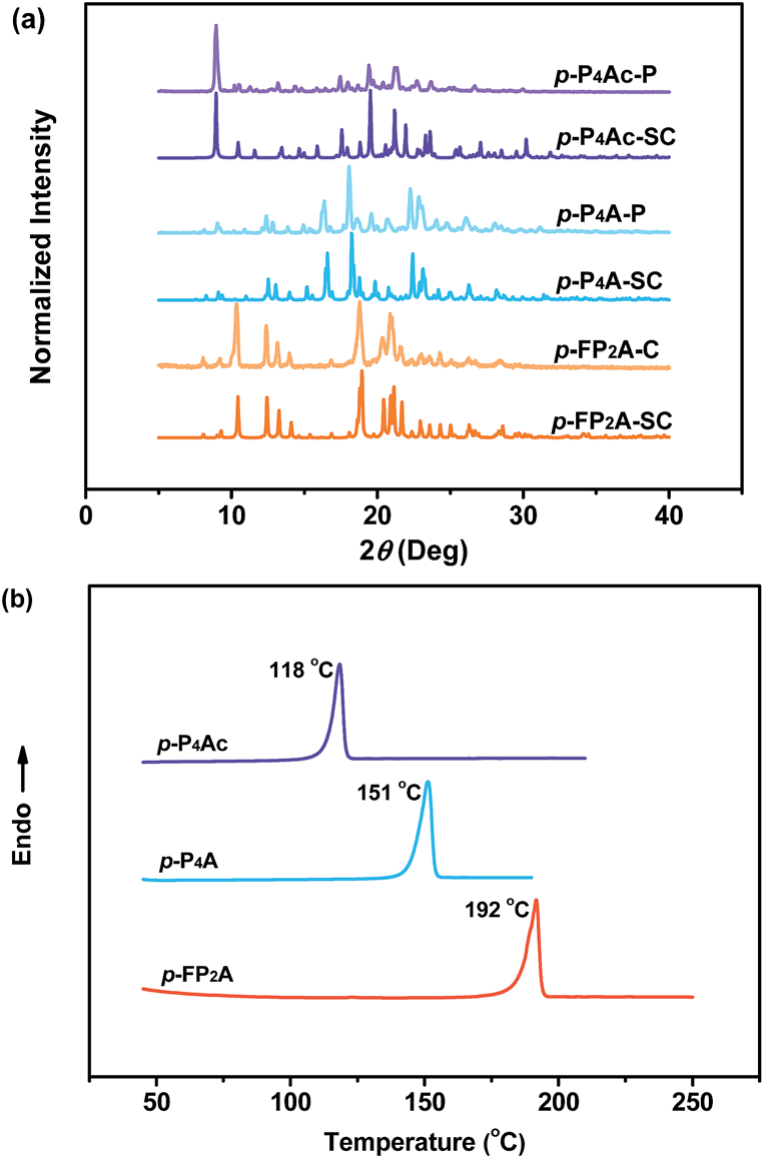
XRD patterns (a) and DSC curves (b) of *p*-P_4_Ac, *p*-P_4_A and *p*-FP_2_A. *p*-P_4_Ac-P, powder of *p*-P_4_Ac; *p*-P_4_Ac-SC, single crystal of *p*-P_4_Ac; *p*-P_4_A-P, powder of *p*-P_4_A; *p*-P_4_A-SC, single crystal of *p*-P_4_A; *p*-FP_2_A-P, powder of *p*-FP_2_A; *p*-FP_2_A-SC, and single crystal of *p*-FP_2_A.

As shown in Fig. 1, the molecular structure of 4-(1,2,2-triphenylvinyl)benzaldehyde (*p*-P_4_A) is simple and directly constructed by a TPE moiety and a formyl group. Under irradiation by UV light, the as-prepared sample of *p*-P_4_A in the solid state emits strong bluish-green light with *λ*_max_ at 487 nm (Fig. 2a). Since the photophysical properties of organic molecules are correlated with their aggregation state, powder X-ray diffraction (XRD) was carried out to determine the phase characteristics of *p*-P_4_A. The sharp peaks in the XRD pattern depicted in Fig. 3a unambiguously illustrate that the as-prepared *p*-P_4_A is crystalline. Moreover, the sample of *p*-P_4_A exhibits no other transition except an intense melting endothermic peak in the differential scanning calorimetry (DSC) curve (Fig. 3b). This result further confirms that the solid powder of *p*-P_4_A is mainly composed of microcrystals that melt at 151 °C. Intriguingly, by shearing the crystals with a spatula in the dark, extremely strong emission was observed without excitation by UV light, confirming the ML character of *p*-P_4_A in the crystalline form (Fig. 2c-I, Video S1†). Indeed, such bright ML of *p*-P_4_A could also be clearly observed by the naked eye even under daylight at room temperature and is maintained while continuously shearing the crystals (Fig. 2c-III and Video S2†). The ML maximum of *p*-P_4_A is at around 487 nm (Fig. 2b), which corresponds well with its photoluminescence (PL). In other words, the PL and ML of *p*-P_4_A result from the same excited state regardless of the excitation modes. Meanwhile, it is worth noting that a ML compound such as *p*-P_4_A is very stable and can be stored in air for more than one year. To further demonstrate the high performance of *p*-P_4_A in ML production, a simple device was fabricated by sandwiching the crystals between a wooden stamper and a piece of glass. As shown in Fig. 2c-II, while driven by mechanical force, the capital letters ‘AITL’ are facilely generated, suggesting that the ML material *p*-P_4_A is promising for displays and optical recording applications.

**Fig. 4:**
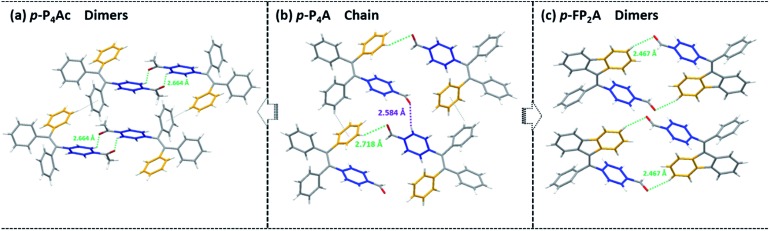
Molecular packing and intermolecular interactions in single crystals of *p*-P_4_Ac (a), *p*-P_4_A (b) and *p*-FP_2_A (c).

Noticeably, considering that both *p*-P_4_A and P_4_TA contain a TPE moiety and a formyl group in their molecular structures, these two units may play a key role in achieving ML properties. To verify this hypothesis, compounds *p*-P_4_Ac and *p*-FP_2_A (Fig. 1) were synthesized to investigate the influence of the TPE moiety and the aldehyde group on ML performance, respectively. As depicted in Fig. 2a and b, although blue-light and green-light with *λ*_max_ at 458 nm and 507 nm were observed for *p*-P_4_Ac and *p*-FP_2_A under UV light irradiation, no ML signal was recorded for their resulting crystals. That is, *p*-P_4_A completely lost its ML activity upon changing the formyl to an acetyl group, or by locking two of the phenyl rings of TPE into the fluorene subunit. To gain more insight into the effects of these two functional fragments, single crystals of *p*-P_4_A, *p*-P_4_Ac and *p*-FP_2_A were isolated by solvent evaporation of their solutions in a mixture of ethanol and CH_2_Cl_2_, and their structures solved by single crystal X-ray diffraction. The simulated XRD patterns from single crystal data of all the three luminophores are almost identical with the experimental data obtained from their crystalline powders (Fig. 3a), implying that the molecular packing modes for the as-prepared samples of *p*-P_4_A, *p*-P_4_Ac and *p*-FP_2_A are similar to the corresponding single crystals.^11^ Further study reveals that the single crystal structure of *p*-P_4_A belongs to the monoclinic system with a non-centrosymmetric polar space group of *P*(2)1 (Table 1). In the unit cells, two crystallographically independent conformations of *p*-P_4_A are found and interact with each other through two different kinds of C–H···O hydrogen bonds (Fig. 4b). As a result, special molecular ‘chains’ are constituted in the crystal structure. Additionally, the molecules and their counter ones show a dihedral angle of 79.68° between the phenyl rings adjacent to the formyl groups, instead of an anti-parallel packing mode, which would favor achieving a non-centrosymmetric molecular arrangement. By contrast, in the cases of *p*-P_4_Ac and *p*-FP_2_A, molecules are parallel or anti-parallel to each other and dimers are formed with the assistance of C–H···O hydrogen bonds (Fig. 4a and c). The highly regular molecular stacking, therefore, largely increases the symmetric elements of the crystal structures, leading to centrosymmetric and non-polar space groups for the single crystals of *p*-P_4_Ac and *p*-FP_2_A (Table 1). Obviously, the synergistic effect of the TPE and the formyl units acts as a crucial factor in yielding a non-centrosymmetric crystal structure and net dipole moment for the remarkable piezoelectric property that promotes the distinct ML emission of *p*-P_4_A by electronic discharge on the crack surface of the microcrystals.4b,^12^

**Table 1:** Single crystal information for the compounds

Compound	Crystal system	Space group	Symmetry	Polarity	ML activity
*p*-P_4_A	Monoclinic	*P*2(1)	Non-centrosymmetric	Polar	Active
*p*-P_4_Ac	Triclinic	*P*1[combining macron]	Centrosymmetric	Non-polar	Inactive
*p*-FP_2_A	Monoclinic	*P*2(1)/*c*	Centrosymmetric	Non-polar	Inactive
*m*-P_4_A	Monoclinic	*P*2(1)	Non-centrosymmetric	Polar	Active
*p*-P_4_A_2_	Orthorhombic	*Pna*2(1)	Non-centrosymmetric	Polar	Active

**Fig. 5:**
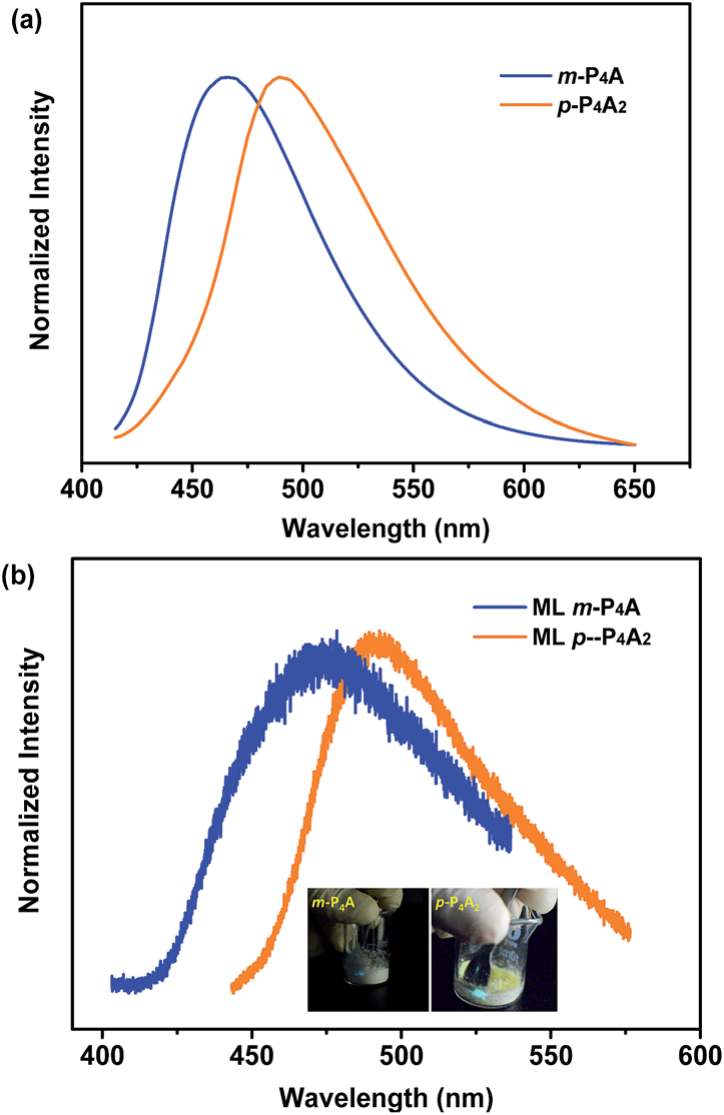
PL (a) and ML (b) spectra of *m*-P_4_A and *p*-P_4_A_2_ in the solid state. The inset of (b) is the images of ML for *m*-P_4_A (left) and *p*-P_4_A_2_ (right). The PL spectra were recorded under excitation of 365 nm UV light.

To further test this proposed principle, two additional luminophores of *m*-P_4_A and *p*-P_4_A_2_ comprising TPE and formyl moieties were synthesized (Fig. 1). Their solid powders were prepared by adding concentrated dichloromethane solutions of the compounds into *n*-hexane under the action of ultrasound. The clear melting transitions at 136 °C and 180 °C in the DSC curves (Fig. S2a†) and the sharp peaks in the XRD patterns (Fig. S2b†) of *m*-P_4_A and *p*-P_4_A_2_ manifest their crystalline morphologies. By varying the substitution position and number of the formyl group(s), *m*-P_4_A and *p*-P_4_A_2_ remain ML active upon grinding or shearing and their emissions are successfully tuned from *λ*_max_ 472 nm to 492 nm (Fig. 5b), which are also close to their corresponding photoluminescence peaks (Fig. 5a). Both *m*-P_4_A and *p*-P_4_A_2_ are also stable and their ML activities could be retained even being stored in air for more than one year. Single crystal structures of these two new ML compounds adopt non-centrosymmetric molecular arrangements with polar space groups of *P*(2)1 and *Pna*(2)1, respectively (Table 1). Similar molecular stacking may thus occur in the solid powders of *m*-P_4_A and *p*-P_4_A_2_ because the experimental XRD patterns of these two compounds almost overlap with the simulated ones from their single crystal data (Fig. S2b†). Careful analysis deciphers that the special molecular ‘chains’ polymerized by the C–H···O hydrogen bonds appear again and the molecules are all at a certain angle to their counter ones in both of the crystal structures (Fig. S3†). These results are in accordance with those observed for *p*-P_4_A, thus attesting that the synergistic effect of TPE and the aldehyde units would disorder the regularity of molecular packing and reduce the symmetry of the crystal structure to provide non-centrosymmetric crystals with ML activity.

Although *m*-P_4_A, *p*-P_4_A_2_ and *p*-P_4_A are all ML active, their performances are different and can be readily distinguished. While *p*-P_4_A_2_ exhibits conspicuous ML even under daylight (Fig. 5b, Video S3 and S4†), the emission of *m*-P_4_A could merely be discerned in dim lighting at room temperature (Video S5†). Meanwhile, it should be noted that the ML brightness of *p*-P_4_A_2_ is slightly weaker than that of *p*-P_4_A. To gain an understanding of this aspect, theoretical calculations based on the ground state geometries in the single crystals were implemented using B3LYP density functional theory (DFT) at the 6-31G(d,p) level to simulate the electronic transition characteristics of the compounds. The results in Fig. S4† show that all the three luminophores present an intermolecular charge transfer (ICT) from the TPE to the phenylaldehyde moiety in the solid state. Furthermore, transitions from HOMO to LUMO for *p*-P_4_A and *p*-P_4_A_2_ and HOMO to LUMO+1 for *m*-P_4_A with large oscillator strength (*f*) over 0.30 were estimated (Table S1†). The dipole moments for the two conformations of *p*-P_4_A are determined to be 4.79 and 5.42 debye (D), which are both larger than that of *p*-P_4_A_2_ (4.78 D) and, especially, of *m*-P_4_A (3.83 D). The higher dipole moment values of the molecules would probably cause a stronger piezoelectric effect when breaking the crystals with a non-centrosymmetric molecular structure, and subsequently result in stronger excitation and higher exciton concentration on the basis of the similarly large oscillator strengths. On the other hand, despite containing the typical AIE moiety of TPE that could eliminate π–π stacking, the emission performances of *m*-P_4_A, *p*-P_4_A_2_ and *p*-P_4_A in the solid state might be diverse from each other. To clarify this issue, the AIE properties were investigated for these three luminophores to demonstrate the effect on ML generation.

**Fig. 6:**
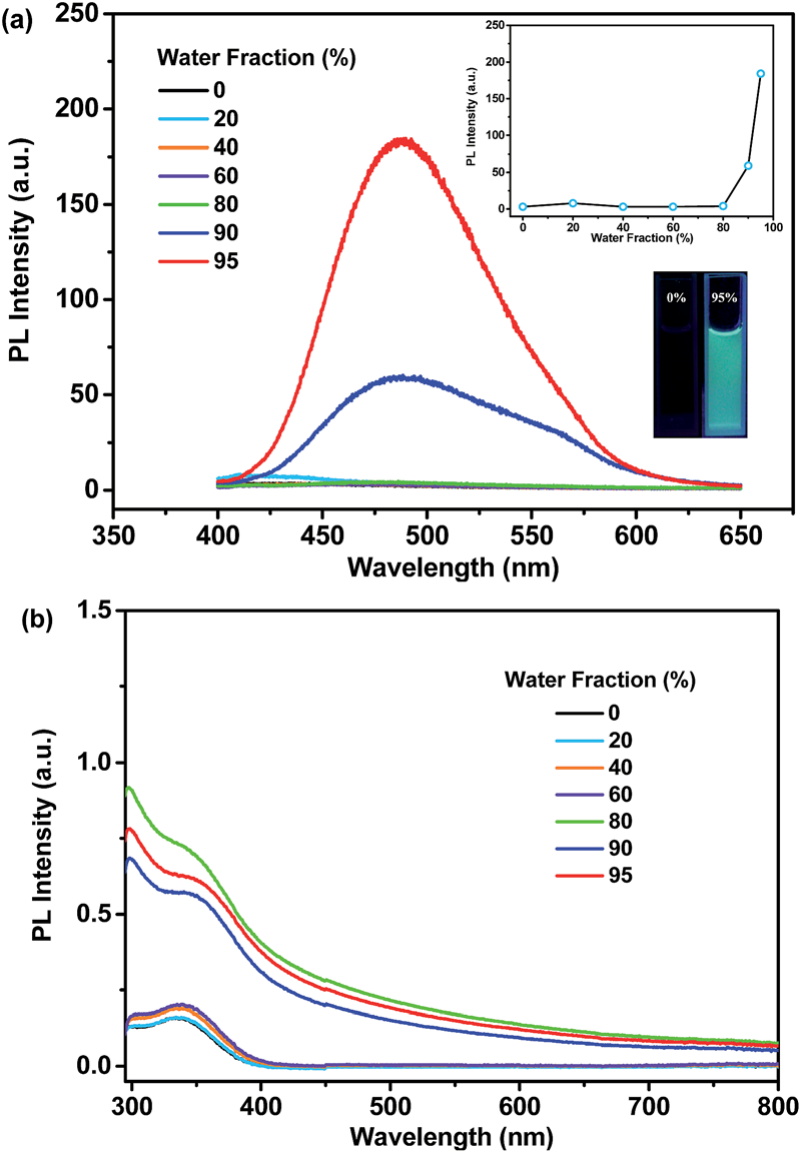
PL (a) and UV-visible (b) spectra of *p*-P_4_A in the mixtures of THF/water with different water content. The inset of (a) shows the changes of peak intensities (upper) of the PL spectra and the fluorescence images (lower) of *p*-P_4_A in pure THF and in the mixture of THF/water with a 95% water fraction. The PL spectra were recorded under excitation of 365 nm UV light.

As shown in Fig. 6a, *p*-P_4_A exhibits extremely weak emission in pure THF solution, where it is well dissolved. Nevertheless, when 95% (v/v) of distilled water is added, significant emission enhancement at around 488 nm is observed and the corresponding PL intensity increased by up to about 54-fold in comparison with the data at 0% water fraction. The UV-visible absorption profile shows a Mie scattering effect for the mixtures of *p*-P_4_A with high water content (Fig. 6b) and particles with an effective diameter (E.D.) of 326 nm are observed in the mixture with 95% water (Table S2 and Fig. S7†), indicating that the emission enhancement of this compound originates from the formation of nanoaggregates.^13^ Evidently, *p*-P_4_A is an excellent AIE chromophore that could effectively suppress non-radiative decay to produce intense light emission in the solid state.^14^ Owing to AIE, the solid powder of *p*-P_4_A affords an impressive fluorescence quantum yield (*Φ*_F,s_) of 0.28, which is higher than most conventional pure organic and organometallic ML compounds. In the single crystal structure of *p*-P_4_A, detrimental species, for instance excimers or exciplexes, caused by π–π stacking interactions have been ruled out due to the highly twisted conformations of the AIE molecules. In addition, the twisted molecular conformations have been immobilized by the multiple molecular interactions of C–H···O and C–H···π to impede the intramolecular rotation and vibration (Fig. 4 and S3†). Accordingly, non-radiative relaxation would be strongly restrained, leading to a notable AIE effect for *p*-P_4_A.

The remarkable emission enhancement emanating from the AIE nature and the prominent piezoelectric effect induced by the large dipolar moment in the non-centrosymmetric molecular structure, therefore makes the ML of *p*-P_4_A highly emissive under the stimulus of mechanical force. Similar results were achieved for the luminophore of *p*-P_4_A_2_ with an emission enhancement of ∼29-fold from pure THF solution to the mixture containing nanoparticles with an E.D. of 396 nm (*λ*_max,PL_ = 486 nm) and a quantum yield of 0.21 in the solid state. Hence, by combining the high dipolar moment value, *p*-P_4_A_2_ could also produce strong ML, which is almost comparable to that of *p*-P_4_A. By contrast, for the *m*-P_4_A system only a slightly increment (∼1.6 times) was recorded for the mixture (*λ*_max,PL_ = 464 nm), indicating its relatively poor AIE property. The *Φ*_F,s_ value of *m*-P_4_A was evaluated to be 0.10, which is moderate among the whole ML material family. As a result, *m*-P_4_A displays weak ML emission at room temperature upon shearing the crystals comprised of low dipolar moment molecules. As compared to the pristine samples, all the three compounds show a dramatic decrease in emission intensity after grinding and the corresponding *Φ*_F,s_ values of *p*-P_4_A, *p*-P_4_A_2_ and *m*-P_4_A are calculated to be 0.12, 0.11 and 0.03, respectively. Although most diffraction peaks which are consistent with the original ones remain observable in the XRD patterns of the ground samples (Fig. S8†), some of the peaks become broader or even disappear. These results corroborate that the molecular packing modes of *p*-P_4_A, *p*-P_4_A_2_ and *m*-P_4_A are partially altered to an amorphous state. On account of the amorphization, intramolecular rotations and vibrations of the phenyl rings of the compounds would probably be enhanced, which therefore largely activate non-radiative decay and reduce the *Φ*_F,s_ values of the ground samples of *p*-P_4_A, *p*-P_4_A_2_ and *m*-P_4_A. Obviously, although the ML activity mainly depends on the crystal symmetry of the compound, its performance would probably be enhanced by creating molecules with larger dipolar moments and more conspicuous AIE properties. From the preceding findings, it seems that all of these requirements could be fulfilled by employing a TPE moiety and one *para*-substituted formyl group as two of the building blocks for this molecular and crystal engineering.

## Conclusions

We have presented a rational design strategy towards very bright ML from purely organic luminophores with AIE properties by introducing a TPE moiety and one *para*-substituted formyl group as the main components of the molecules. Under the synergistic interplay of these two units, a non-centrosymmetric crystal structure, larger molecular dipolar moment and more notable AIE performance are successfully achieved to produce a significantly enhanced ML emission upon the stimulus of mechanical force. Further studies will focus on the development of more efficient ML luminogens following this precise design strategy and the applications of ML materials in optical recording and pressure sensing.

## Supplementary Material

Supplementary information

Crystal structure data
