# Supplementary material for: Achieving very bright mechanoluminescence from purely organic luminophores with aggregation-induced emission by crystal design
Source: Chem Sci. 2016 Apr 26;7(8):5307–12. doi: 10.1039/c6sc01325b (PMC6020548; doi:10.1039/c6sc01325b)
Supplement: Supplementary file 1 [file SC-007-C6SC01325B-s001.pdf]

Electronic Supplementary Material (ESI) for Chemical Science.
This journal is © The Royal Society of Chemistry 2016

Electronic Supplementary Information

Achieving Very Bright Mechanoluminescence from Purely Organic Luminophores with Aggregation-Induced Emission by Crystal Design

Bingjia Xu,[a,b] Wenlang Li,[a] Jiajun He,[a] Sikai Wu,[a] Qiangzhong Zhu,[b] Zhiyong Yang,*[a] Yuan-Chun Wu,[c] Yi Zhang,*[a] Chongjun Jin,[b] Po-Yen Lu,[c] Zhenguo Chi,*[a] Siwei Liu,[a] Jiarui Xu[a] and Martin R. Bryce[d]

[a] *PCFM Lab, GD HPPC Lab, Guangdong Engineering Technology Research Center for High-performance Organic and Polymer Photoelectric Functional Films, State Key Laboratory of Optoelectronic Material and Technologies, School of Chemistry and Chemical Engineering, Sun Yet-sen University, Guangzhou 510275, China.*

E-mail: yangzhy29@mail.sysu.edu.cn; ceszy@mail.sysu.edu.cn; chizhg@mail.sysu.edu.cn; Tel: +86 20 84112712.

[b] *State Key Laboratory of Optoelectronic Material and Technologies, School of Physics and Engineering, Sun Yat-sen University, Guangzhou 510275, China.*

[c] *Shenzhen China Star Optoelectronics Technology Co., Ltd, Guangdong, China.*

[d] *Department of Chemistry, Durham University, Durham DH1 3LE, UK.*

1. General experimental procedures

Materials (2-bromoethene-1,1,2-triyl)tribenzene, 9*H*-fluoren-9-one, benzophenone, tetrakis(triphenyl phosphine) palladium(0), Aliquat 336, (4-acetylphenyl)boronic acid, (4-formylphenyl)boronic acid and (3-formylphenyl)boronic acid purchased from Alfa Aesar were used as received. The compounds 4-(1,2,2-triphenylvinyl)benzaldehyde (*p*-P_4A)[1] and 9-(dibromomethylene)-9*H*-fluorene (FBr_2)[2], and (2,2-dibromo ethene-1,1-diyl)dibenzene (P_2Br_2)[2] were synthesized according to the literature methods. Ultra-pure water was used in the experiments. All other reagents and solvents were purchased with analytical grade from Guangzhou Jincheng Company (China) and used without further purification. The water/tetrahydrofuran mixtures with different water fractions were prepared by slowly adding distilled water into the THF solution of the samples under ultrasound at room temperature.

Characterization Proton and carbon NMR (^{1}HNMR and ^{13}CNMR) spectra were measured on a Mercury-Plus 300 spectrometer, a Bruker AVANCE 400 spectrometer or a Bruker AVANCE III spectrometer ($CDCl_3$, tetramethylsilane as the internal standard). The mass spectra were measured using Thermo spectrometers (DSQ & MAT95XP-HRMS). The FT-IR spectra were obtained on a Nicolet NEXUS 670 spectrometer (KBr pellet). The elemental analysis was performed with a Vario EL analyzer. The UV-visible absorption spectra were determined on a Hitachi U-3900 spectrophotometer. The PL spectra were measured on an Ocean Optics Maya Pro2000 instrument with a 365 nm Ocean Optics LLS-LED as the excitation source. Light was introduced into the detector through an optical fiber. The ML spectra were collected from an Acton SP2750 spectrometer with a liquid-nitrogen-cooled CCD (SPEC-10, Princeton) as a power detector. ML images of *p*-P_4A, *m*-P_4A and *p*-P_4A_2 were directly extracted from the supplementary movies (Videos S1, S2, S4 and S5) captured by a digital camera (Nikon D5100) with an AF-S Nikkor 35mm f/1.8G DX camera lens. This method is similar to the one employed in previous report.3 Herein, the movies were transcribed by the camera in

an automatic mode with a capturing speed of 29 frames per second. The image of the capital letters "AITL" was captured by the Nikon D5100 digital camera in a manual mode. The corresponding parameters were set up as follows: aperture value f/2, exposure time 26 s, ISO 1600 and exposure compensation +0.3. The thermal behaviors were determined by DSC at heating and cooling rates of 10 °C/min under N_2 atmosphere using a NETZSCH thermal analyzer (DSC 204F1). Wide-angle XRD measurements were performed at 293 K using a Bruker X-ray diffractometer (D8 ADVANCE, Germany) with an X-ray source of Cu Kα (λ= 0.15406 nm) at 40 kV and 40 mA at a scan rate of 4° (2ϑ)/min. The fluorescence quantum yields of solid powders were measured in air on an integrating sphere (HAMAMATSU C11347) with a 330 nm UV light as the excitation source. The quantum chemistry calculations were performed at the B3LYP/6-31G (d, p) level of theory using the DFT method in the Gaussian 09 software.

The single crystals of all the target compounds were isolated from the mixtures of ethanol and CH_2Cl_2 through the method of solvent evaporation. X-ray diffraction data for the single crystals were collected from a Bruker Smart 1000 CCD with Cu-Kα radiation (λ =1.54178 Å) at 150(10) K. All the structures were solved using direct methods following the difference Fourier syntheses. All non-hydrogen atoms were anisotropically refined through least-squares on F^2 using the SHELXTL program suite. The anisotropic thermal parameters were assigned to all non-hydrogen atoms. The hydrogen atoms attached to carbon were placed in idealized positions and refined using a riding model to the atom from which they were attached. The pictures of the structures were produced using Diamond 3.2. CCDC 1468361, 1468362, 1468363, 1468364, and 1468365 contain the supplementary crystallographic data of *m*-P_4A, *p*-FP_2A, *p*-P_4A, *p*-P_4A_2, *p*-P_4Ac for this paper, respectively.

References

1 X. Q. Zhang, Z. G. Chi, H. Y. Li, B. J. Xu, X. F. Li, W. Zhou, S. W. Liu, Y. Zhang and J. R. Xu., *Chem.-Asian J.* 2011, **6**, 808.

2 P. M. Donovan and L. T. Scott., *J. Am. Chem. Soc.*, 2003, **126**, 3108.

3 S. M. Jeong, S. Song, S. Lee, and N. Y. Ha., *Adv. Mater.*, 2013, **25**, 6194.

2. Synthesis

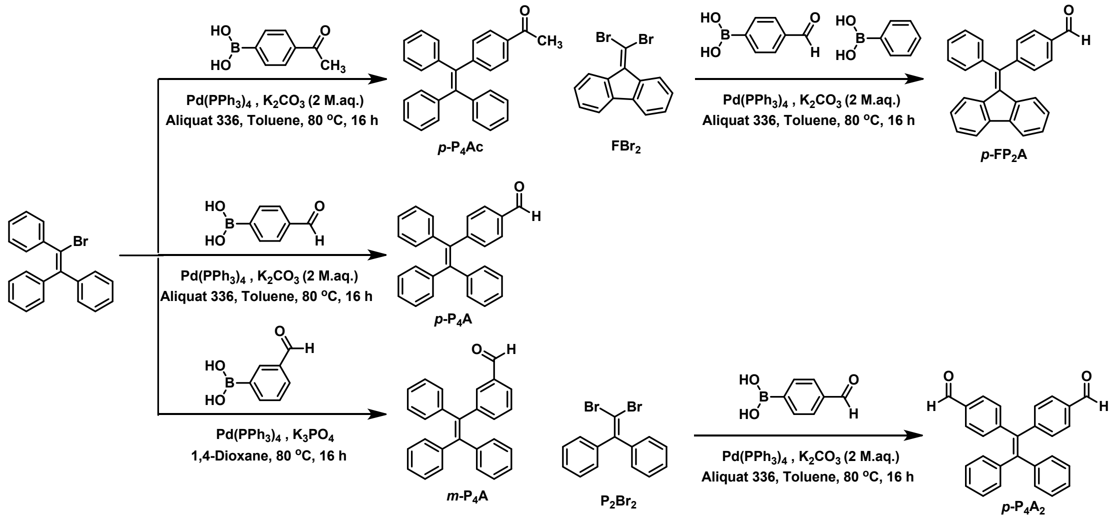

Scheme 1. Synthetic routes of the target compounds.

Synthesis of 4-(1,2,2-triphenylvinyl)benzaldehyde (p-P_4A) [1] (2-bromoethene-1,1,2-triyl)tribenzene (1.00 g, 2.98 mmol) and (4-formylphenyl)boronic acid (0.49 g, 3.28 mmol) were dissolved in toluene (30 mL), and then 2 M aqueous K_2CO_3 solution (4.5 mL) and Aliquat 336 (5 drops) were added. The mixture was stirred for 40 min under an argon atmosphere at room temperature. Then the $Pd(PPh_3)_4$ catalyst was added, and the reaction mixture was stirred at 80 ˚C for 16 h. After cooling to room temperature, the product was concentrated and purified by silica gel column chromatography with dichloromethane/*n*-hexane (v/v=1:3). Compound *p*-P_4A was obtained as a yellow crystalline solid in 97% yield (1.04 g). ^{1}H NMR (300 MHz, $CDCl_3$) δ (ppm): 9.90-9.87 (s, 1 H); 7.64-7.57 (d, *J*=8.4 Hz, 2 H); 7.22-7.16 (d, *J*=8.1 Hz, 2 H); 7.15-7.05 (m, 9 H); 7.05-6.96 (m, 6 H). EI-MS, *m/z*: $[M]^+$ 360; calcd for $C_{27}H_{20}O$ 360. HRMS, *m/z*: $[M]^+$ 360.1511; calcd for $C_{27}H_{20}O$ 360.1514. Anal. Calc. for $C_{27}H_{20}O$: C 89.97%, H 5.59%; found: C 89.91%, H 5.63%.

Synthesis of 1-(4-(1,2,2-triphenylvinyl)phenyl)ethanone (p-P_4Ac) (2-bromoethene-1,1,2-triyl) tribenzene (1.00g, 2.98 mmol) and (4-acetylphenyl)boronic acid (0.54 g, 3.28 mmol) were dissolved in toluene (30 mL), and then 2 M aqueous K_2CO_3 solution (4.5 mL) and Aliquat 336 (5 drops) were added. The mixture was stirred for 40 min under an argon atmosphere at room temperature. Then the $Pd(PPh_3)_4$ catalyst was added and the reaction mixture was stirred at 80 ˚C for 16 h. After cooling to room temperature, the product was concentrated and purified by silica gel column chromatography with dichloromethane/*n*-hexane (v/v=1:3). *p*-P_4Ac was obtained as a white crystalline solid in 94% yield (1.05 g). ^{1}H NMR (300 MHz, $CDCl_3$) δ (ppm): 7.72-7.64 (d, *J*=8.1 Hz, 2 H); 7.16-7.06 (m, 11 H); 7.05-6.95 (m, 6 H); 2.57-2.50 (s, 3 H). ^{13}C NMR (100 MHz, $CDCl_3$) δ (ppm): 191.79, 146.66, 144.20, 143.55, 143.50, 141.69, 140.19, 137.29, 135.09, 132.00, 131.35, 131.31, 130.34, 130.18, 128.02, 127.81, 127.76, 127.66, 127.34, 126.64, 126.59, 126.55, 126.50. FT-IR (KBr) υ (cm^{-1}): 3056, 3022, 1675, 1600, 1490, 1441, 1263, 763, 699. EI-MS, m/z: $[M]^+$ 374; calcd for $C_{28}H_{22}O$ 374. HRMS, m/z: $[M]^+$ 374.1662; calcd for $C_{28}H_{22}O$ 374.1671. Anal. Calc. for $C_{28}H_{22}O$: C 89.81%, H 5.92%; found: C 89.84%, H 5.96%.

Synthesis of 4-((9H-fluoren-9-ylidene)(phenyl)methyl)benzaldehyde (p-FP_2A) FBr_2 (1.00g, 2.97mmol), benzeneboronic acid (0.40g, 3.27mmol) and (4-formylphenyl)boronic acid (0.49g, 3.27mmol) were dissolved in toluene (30 mL), and then 2 M aqueous K_2CO_3 solution (4 mL) and Aliquat 336 (5 drops) were added. The mixture was stirred for 40 min under an argon atmosphere at room temperature. Then the $Pd(PPh_3)_4$ catalyst was added and the reaction mixture was stirred at 80 ˚C for 16 h. After cooling to room temperature, the product was concentrated and purified by silica gel column chromatography with dichloromethane/*n*-hexane (v/v=1:2). *p*-FP_2A was obtained as a pale yellow crystalline solid in 46% yield (0.49 g). ^{1}H NMR (300 MHz, $CDCl_3$) δ (ppm): 10.13-10.00 (s, 1 H); 7.98-7.88 (d, *J*=8.0 Hz, 2 H); 7.73-7.65 (d, *J*=7.5 Hz, 2 H); 7.61-7.54 (d, *J*=8.3 Hz, 2 H); 7.46-7.40 (m, 3 H); 7.39-7.34 (m, 2 H); 7.29-7.21 (t, 2 H); 6.96-6.89 (m, 2 H); 6.64-6.58 (d, *J*=8.0 Hz, 2 H). ^{13}C NMR (100 MHz, $CDCl_3$) δ (ppm): 191.76, 149.22, 143.31, 142.12, 140.83, 140.67, 138.35, 138.08, 135.80, 135.31, 130.51, 130.18, 129.65, 129.06, 128.55, 128.18, 126.64, 126.56, 125.05, 124.84, 119.48, 119.36. FT-IR (KBr) υ (cm^{-1}): 3052, 2841, 1700, 1598, 1437, 737, 698. EI-MS, m/z: $[M]^+$ 358; calcd for $C_{27}H_{18}O$ 358. HRMS, m/z: $[M]^+$ 358.1354; calcd for $C_{27}H_{18}O$ 358.1358. Anal. Calc. for $C_{27}H_{18}O$: C 90.47%, H 5.06%; found: C 90.42%, H 5.09%.

Synthesis of 3-(1,2,2-triphenylvinyl)benzaldehyde (*m*-P_4A) (2-bromoethene-1,1,2-triyl)tribenzene (1.23 g, 3.67 mmol) and (3-formylphenyl)boronic acid (0.50 g, 3.33 mmol) were dissolved in 1,4-dioxane (30 mL), and then potassium phosphate (1.72g, 8.10 mmol) were added. The mixture was stirred for 40 min under an argon atmosphere at room temperature. Then the $Pd(PPh_3)_4$ catalyst was added and the reaction mixture was stirred at

80 ˚C for 16 h. After cooling to room temperature, the product was concentrated and purified by silica gel column chromatography with dichloromethane/*n*-hexane (v/v=1:4). *m*-P_4A was obtained as a white crystalline solid in 72% yield (0.70 g). ^1H NMR (300 MHz, $CDCl_3$) δ (ppm): 9.81-9.76 (s, 1 H); 7.63-7.59 (dd, 1 H); 7.54-7.49 (s, 1 H); 7.33-7.21 (m, 2 H); 7.15-7.07 (m, 9 H), 7.06-6.99 (m, 6 H). ^{13}C NMR (100 MHz, $CDCl_3$) δ (ppm): 192.20, 144.83, 143.10, 143.03, 142.86, 142.43, 139.45, 137.28, 136.12, 133.05, 131.26, 131.23, 131.15, 128.37, 127.90, 127.87, 127.72, 127.22, 126.82, 126.74. FT-IR (KBr) υ (cm^{-1}): 3051, 2716, 2795, 1702, 1586, 1437, 755, 700. EI-MS, *m/z*: $[M]^+$ 360; calcd for $C_{27}H_{20}O$ 360. HRMS, *m/z*: $[M]^+$ 360.1508; calcd for $C_{27}H_{20}O$ 360.1514. Anal. Calc. for $C_{27}H_{20}O$: C 89.97%, H 5.59%; found: C 89.93%, H 5.61%.

Synthesis of 4,4'-(2,2-diphenylethene-1,1-diyl)dibenzaldehyde (p-P_4A_2) P_2Br_2 (1.00g, 2.97mmol) and (4-formylphenyl)boronic acid (0.98 g, 6.51 mmol) were dissolved in toluene (30 mL), and then 2 M aqueous K_2CO_3 solution (8 mL) and Aliquat 336 (5 drops) were added. The mixture was stirred for 40 min under an argon atmosphere at room temperature. Then the $Pd(PPh_3)_4$ catalyst was added and the reaction mixture was stirred at 80 ˚C for 16 h. After cooling to room temperature, the product was concentrated and purified by silica gel column chromatography with dichloromethane/n-hexane (v/v=2:1). *p*-P_4A_2 was obtained as a yellow crystalline solid in 83% yield (1.09 g). ^1H NMR (500 MHz, $CDCl_3$) δ (ppm): 9.93-9.90 (s, 2 H); 7.66-7.63 (d, *J*=8.3 Hz, 4 H); 7.19-7.11 (m, 10 H); 7.04-6.99 (dd, *J*=7.9, 1.5 Hz, 4 H). ^{13}C NMR (125 MHz, $CDCl_3$) δ (ppm): 191.75, 149.62, 145.08, 142.37, 138.52, 134.59, 131.39, 131.21, 129.37, 128.03, 127.53.. FT-IR (KBr) υ (cm^{-1}): 3058, 2800, 2744, 1694, 1597, 1564, 1208, 1166, 700. EI-MS, *m/z*: $[M]^+$ 388; calcd for $C_{28}H_{20}O_2$ 388. HRMS, *m/z*: $[M]^+$ 388.1454; calcd for $C_{28}H_{20}O_2$ 388.1463. Anal. Calc. for $C_{28}H_{20}O_2$: C 86.57%, H 5.19%; found: C 86.52%, H 5.23%.

3. Figures and Tables

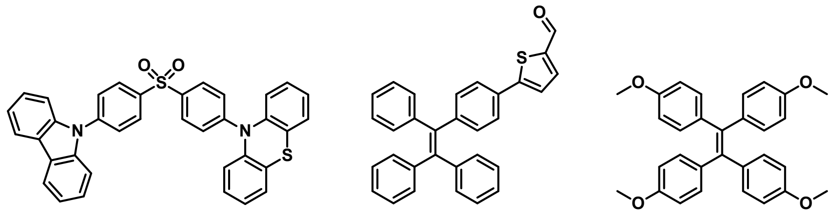

SFPC P_4TA TMPE

a   b   c

Fig. S1 Molecular structures of previously reported AIE-ML luminophores.

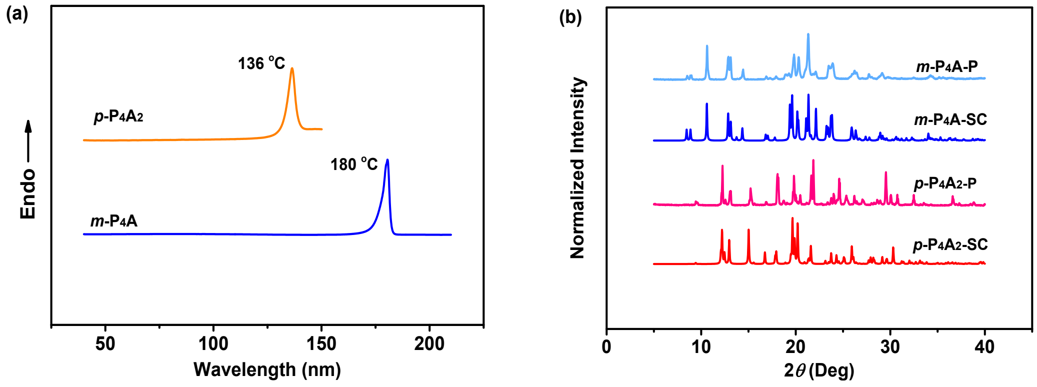

Fig. S2 DSC curves (a) and XRD patterns (b) of *m*-P_4A and *p*-P_4A_2. *m*-P_4A-P, powder of *m*-P_4A; *m*-P_4A-SC, single crystal of *m*-P_4A; *p*-P_4A_2-P, powder of *p*-P_4A_2; *p*-P_4A_2-SC, single crystal of *p*-P_4A_2.

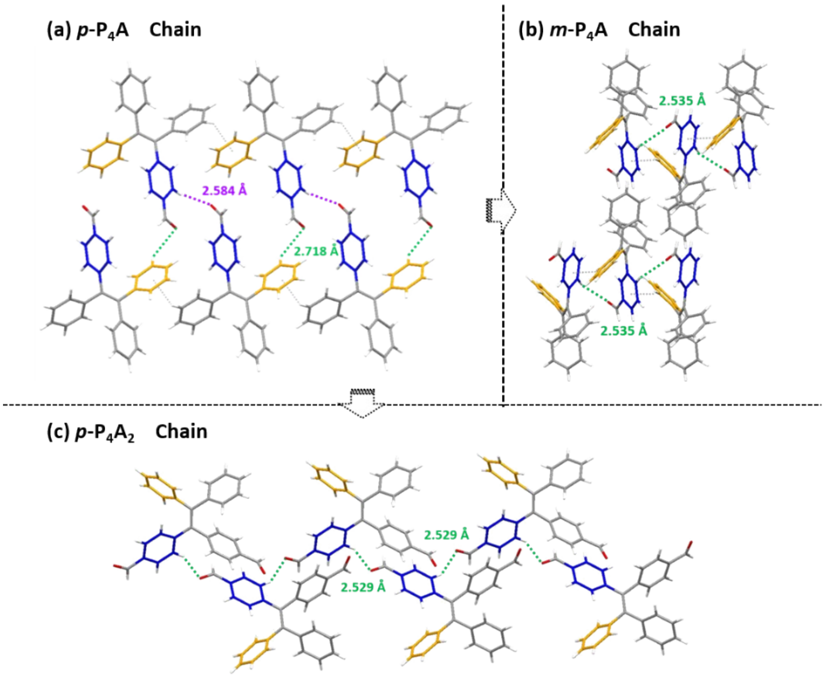

Fig. S3 Molecular packing and intermolecular interactions of *p*-P_4A (a), *m*-P_4A (b) and *p*-P_4A_2 (c) in their single crystal structures

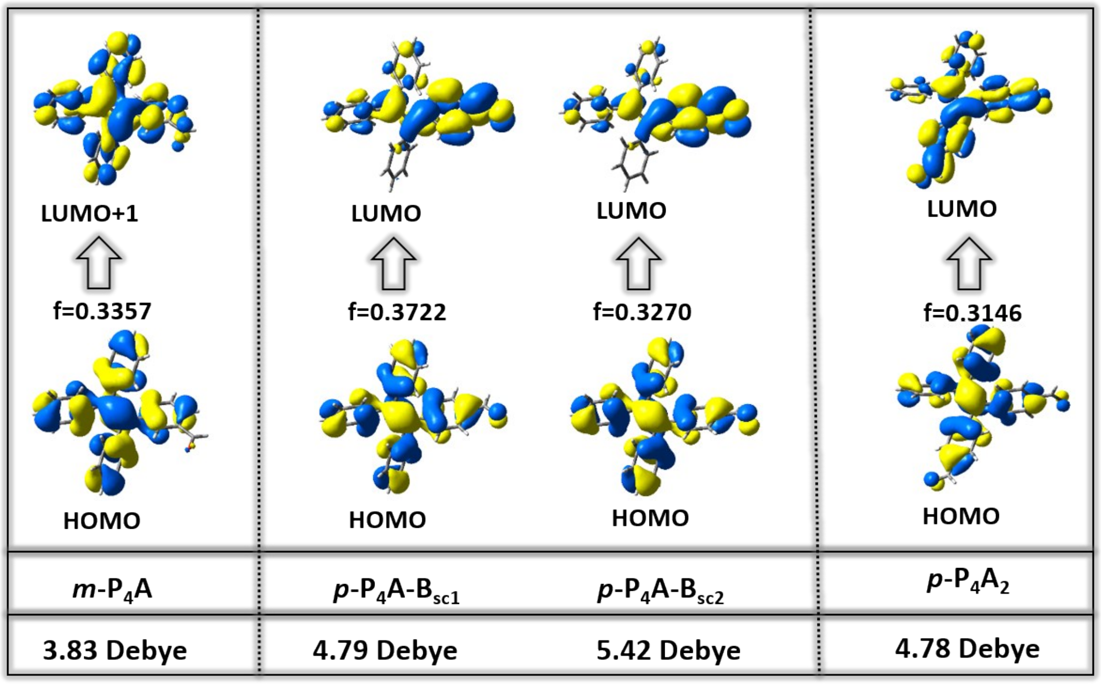

Fig. S4 The HOMO (lower images), LUMO (upper images), oscillator strengths (f) and dipolar moments of the molecules in their single crystal structures. *p*-P_4A-B_{SC1} and *p*-P_4A-B_{SC2} are the two conformations of *p*-P_4A.

Table S1. Computed vertical transitions and their oscillator strengths.

Conformation	Oscillator Strength (*f*)	Transition	Coefficients
p-P_4A-B_{SC1} [a]	0.3722	HOMO→LUMO	0.70088
	0.0020	HOMO-2→LUMO	0.56951
		HOMO-2→LUMO+1	0.16973
		HOMO-1→LUMO	-0.34159
	0.1340	HOMO-3→LUMO	0.14718
		HOMO-1→LUMO	-0.10956
		HOMO→LUMO+1	0.66590
	0.0233	HOMO-3→LUMO	0.15269
		HOMO-2→LUMO	0.32352
		HOMO-1→LUMO	0.56620
		HOMO→LUMO+1	0.10796
		HOMO→LUMO+2	0.11371
p-P_4A-B_{SC2} [a]	0.0004	HOMO-1→LUMO	0.67912
		HOMO-1→LUMO+1	-0.14246
	0.3270	HOMO→LUMO	0.69801
	0.0939	HOMO-2→LUMO	0.49176
		HOMO→LUMO+1	0.48536
	0.1160	HOMO-3→LUMO	0.36658
		HOMO-2→LUMO	0.42717
		HOMO→LUMO+1	-0.39175
m-P_4A	0.0152	HOMO→LUMO	0.69946
	0.0007	HOMO-4→LUMO	0.67992
		HOMO-4→LUMO+1	0.10950
	0.3357	HOMO→LUMO+1	0.70038
	0.0157	HOMO-2→LUMO	-0.11298
		HOMO-1→LUMO	0.61379
		HOMO-1→LUMO+1	-0.14060
		HOMO-1→LUMO+2	-0.25398
p-P_4A_2	0.3146	HOMO→LUMO	0.68955
		HOMO→LUMO+1	-0.13006
	0.2413	HOMO→LUMO	0.12768
		HOMO→LUMO+1	0.68562
	0.0000	HOMO-3→LUMO	-0.12430
		HOMO-2→LUMO	0.48977
		HOMO-2→LUMO+1	0.34096
		HOMO-2→LUMO+3	0.14894
		HOMO-1→LUMO	-0.21991
		HOMO-1→LUMO+1	-0.14811
	0.0020	HOMO-4→LUMO	-0.37880
		HOMO-4→LUMO+1	0.51392

	HOMO-3→LUMO	0.12952
	HOMO-3→LUMO+1	-0.17444

[a] p-P_4A-B_{SC1} and p-P_4A-B_{SC2} are the two conformations of p-P_4A.

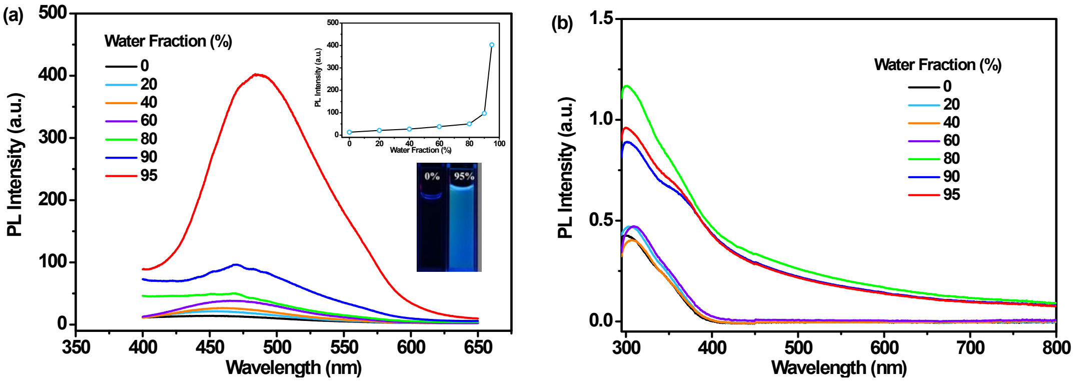

Fig. S5 PL (a) and UV-visible (b) spectra of p-P_4A_2 in the mixtures of THF/water with different water contents. The inset of (a) are the changes of peak intensities (upper) of the PL spectra and the fluorescence images (lower) of p-P_4A_2 in pure THF and in the mixtures of THF/water with 95% water fraction. The PL spectra were recorded under the excitation of 365 nm UV light.

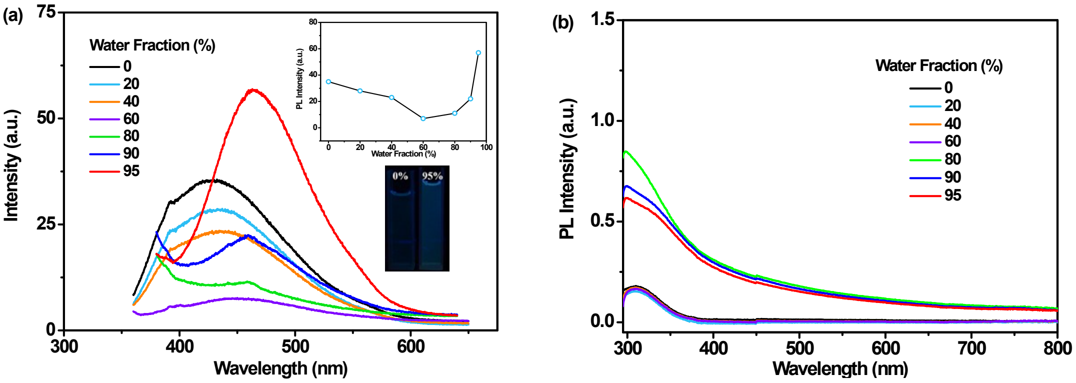

Fig. S6 PL (a) and UV-visible (b) spectra of m-P_4A in the mixtures of THF/water with different water contents. The inset of (a) are the changes of peak intensities (upper) of the PL spectra and the fluorescence images (lower) of m-P_4A in pure THF and in the mixtures of THF/water with 95% water fraction. The PL spectra were recorded under the excitation of 365 nm UV light.

Table S2 Effective diameter and polydispersity of the compounds in THF/water mixtures.[a]

Compound	Effective Diameter (nm)	Polydispersity
p-P_4A	326.36	0.161
p-P_4A_2	395.68	0.176
m-P_4A	339.06	0.179

[a] In THF/water mixtures with 95% water content.

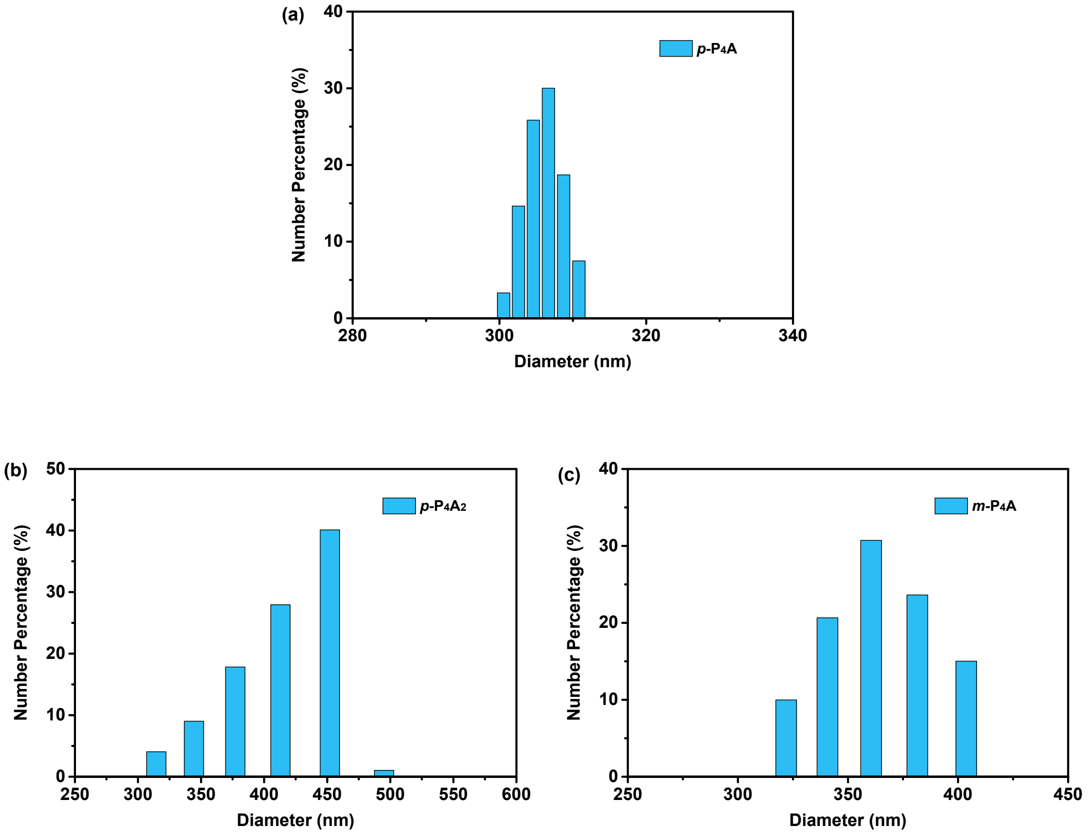

Fig. S7 The particle diameter distributions of the compounds in THF/water mixtures with 95% water content.

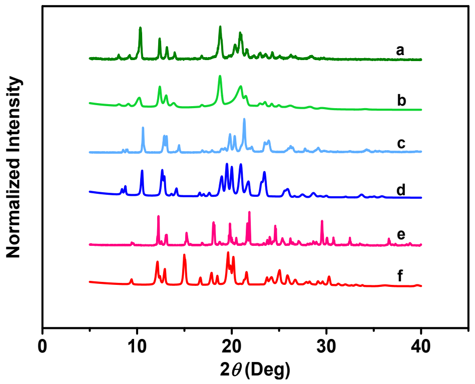

Fig. S8 XRD patterns of the original and ground samples of the compounds. a, Original sample of *p*-P_4A; b, ground sample of *p*-P_4A; c, original sample of *m*-P_4A; d, ground sample of *m*-P_4A; e, original sample of *p*-P_4A_2; f, ground sample of *p*-P_4A_2.

4. Structural Information

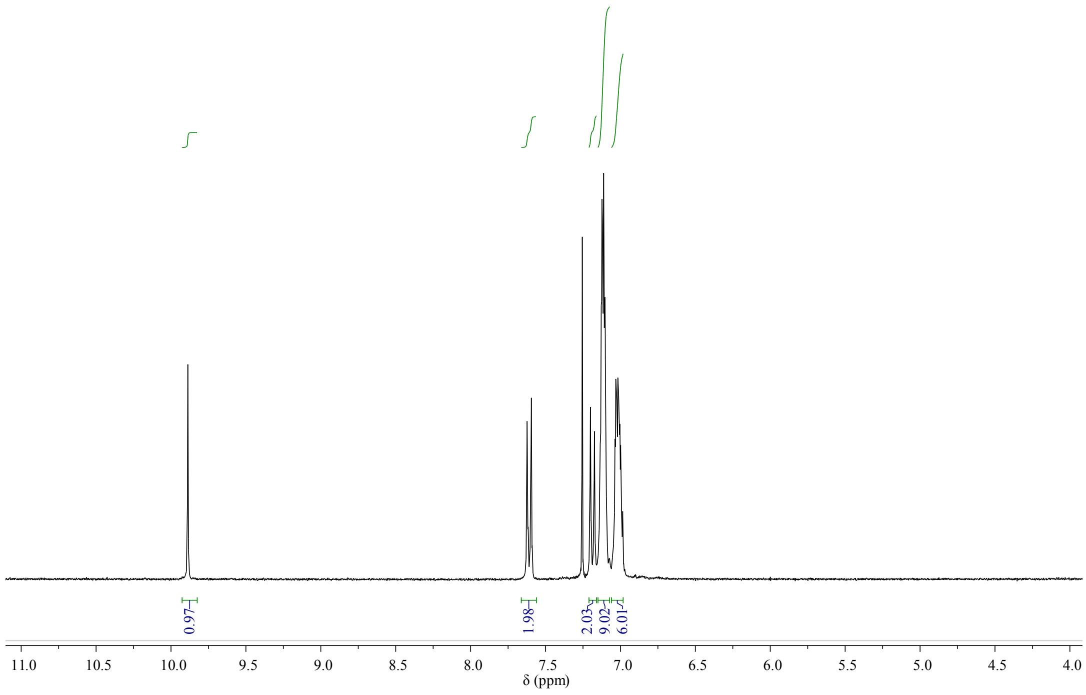

Fig. S9 ^{1}H NMR spectrum of *p*-P_4A in $CDCl_3$

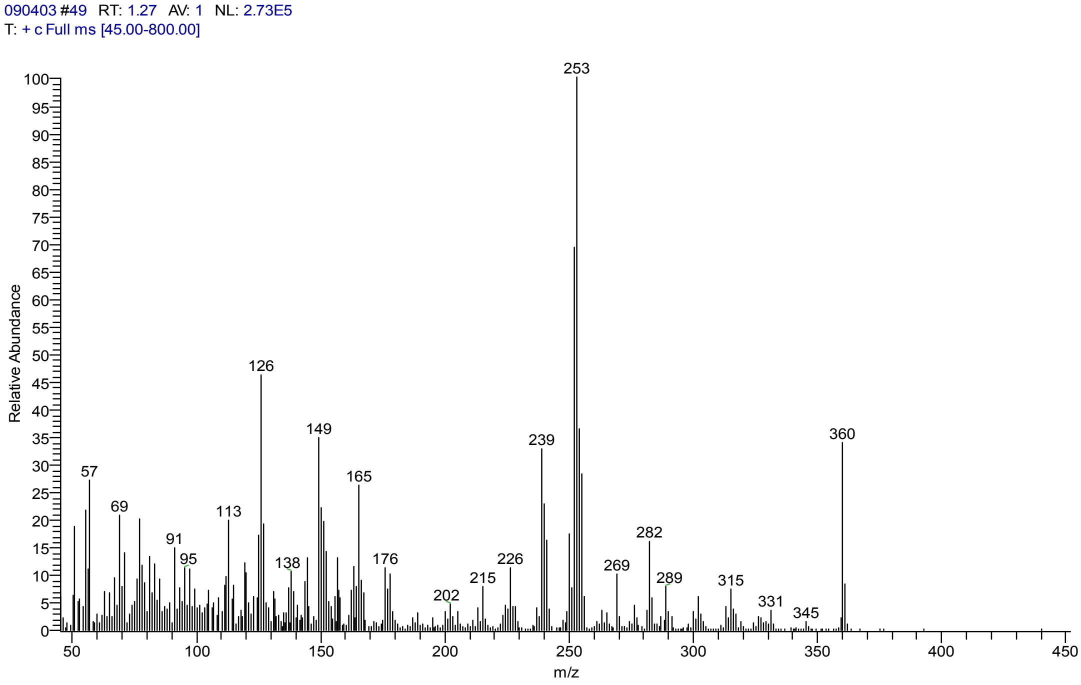

Fig. S10 EI-MS of *p*-P_4A

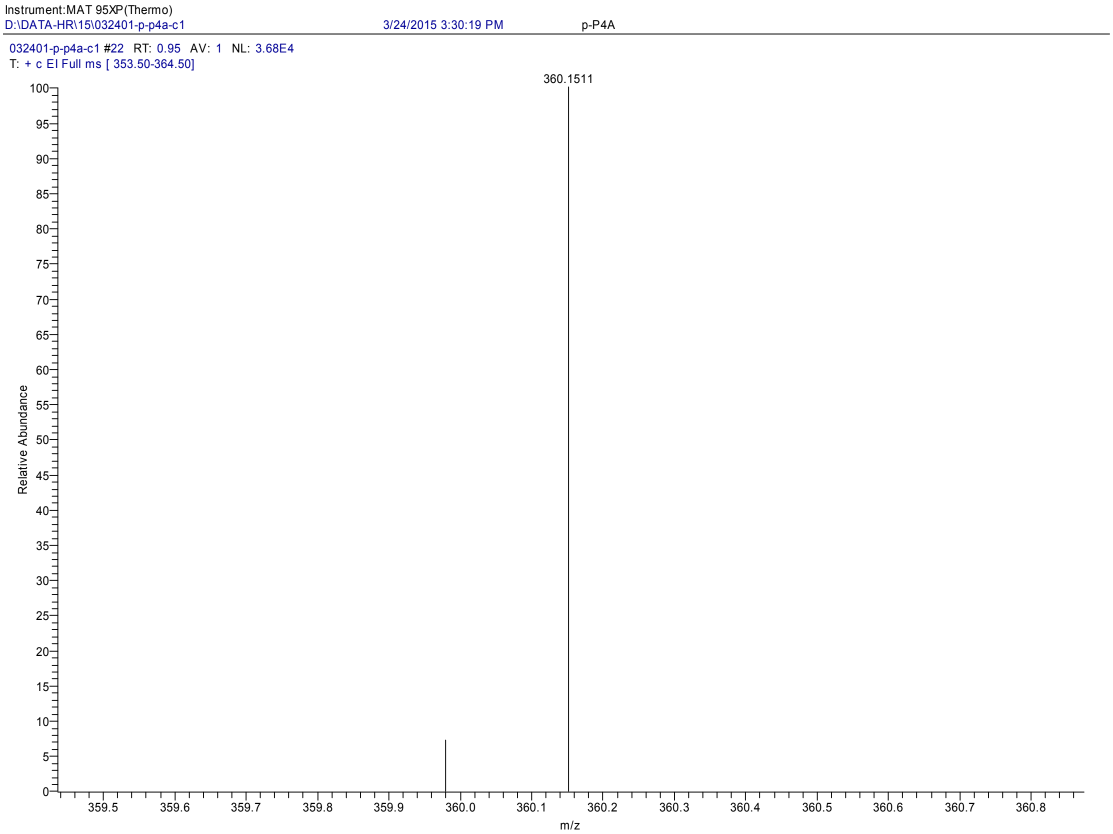

Fig. S11 HRMS of *p*-P_4A

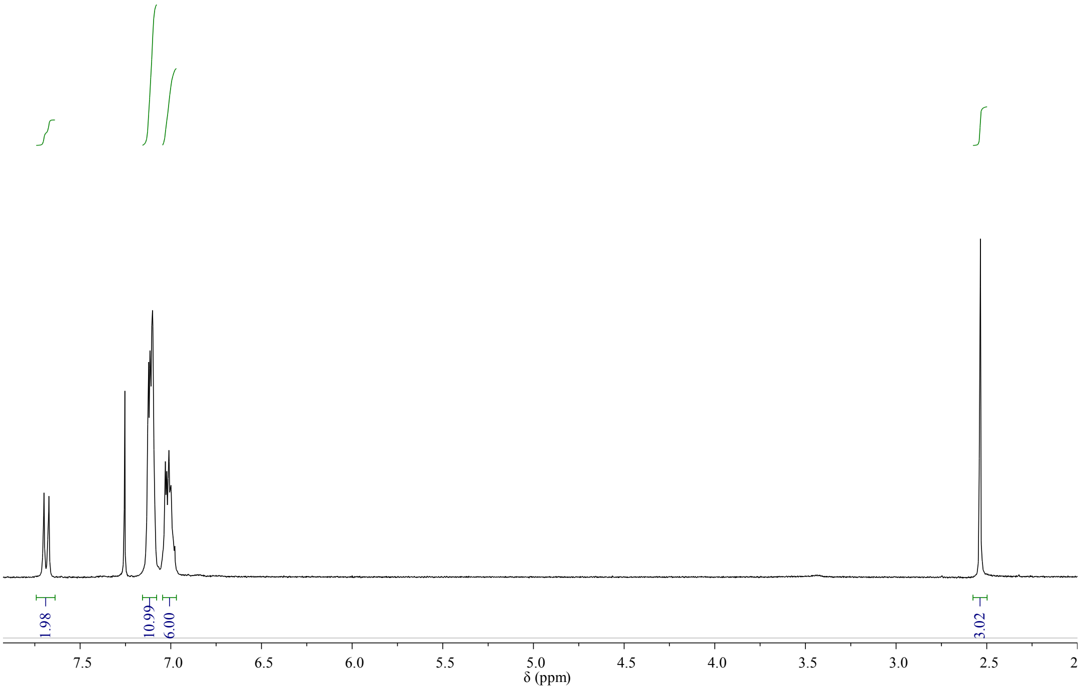

Fig. S12 ^{1}H NMR spectrum of *p*-P_4Ac in $CDCl_3$

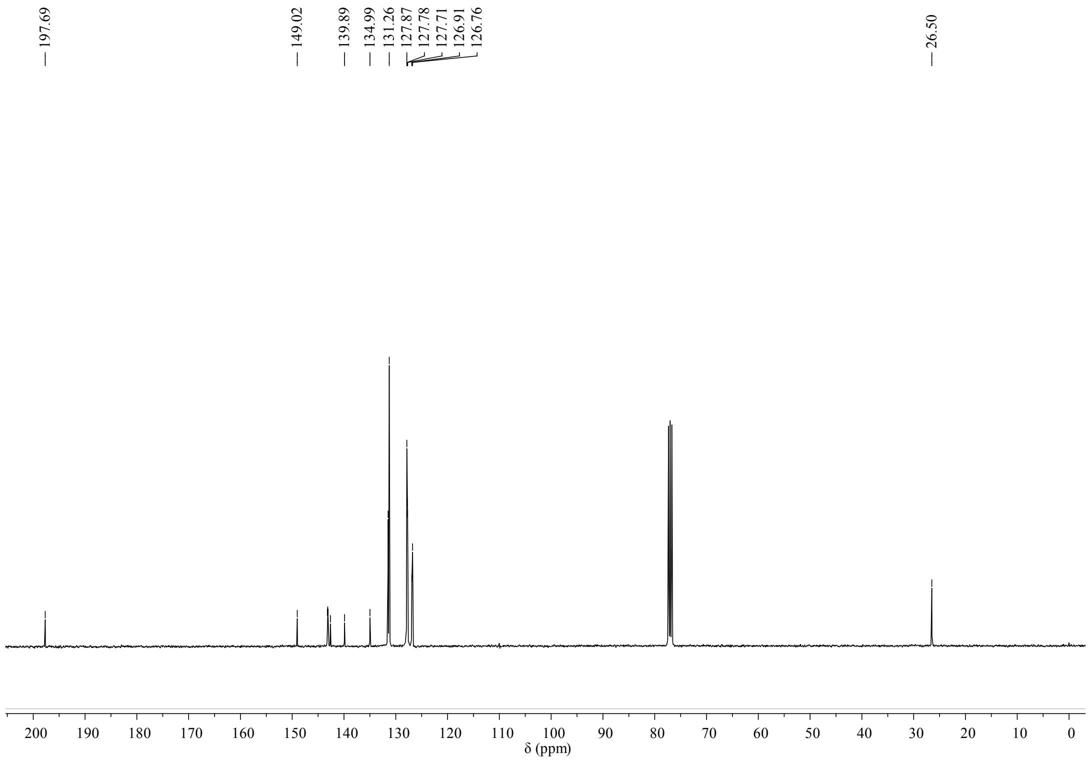

Fig. S13 ^{13}C NMR spectrum of *p*-P_4Ac in $CDCl_3$

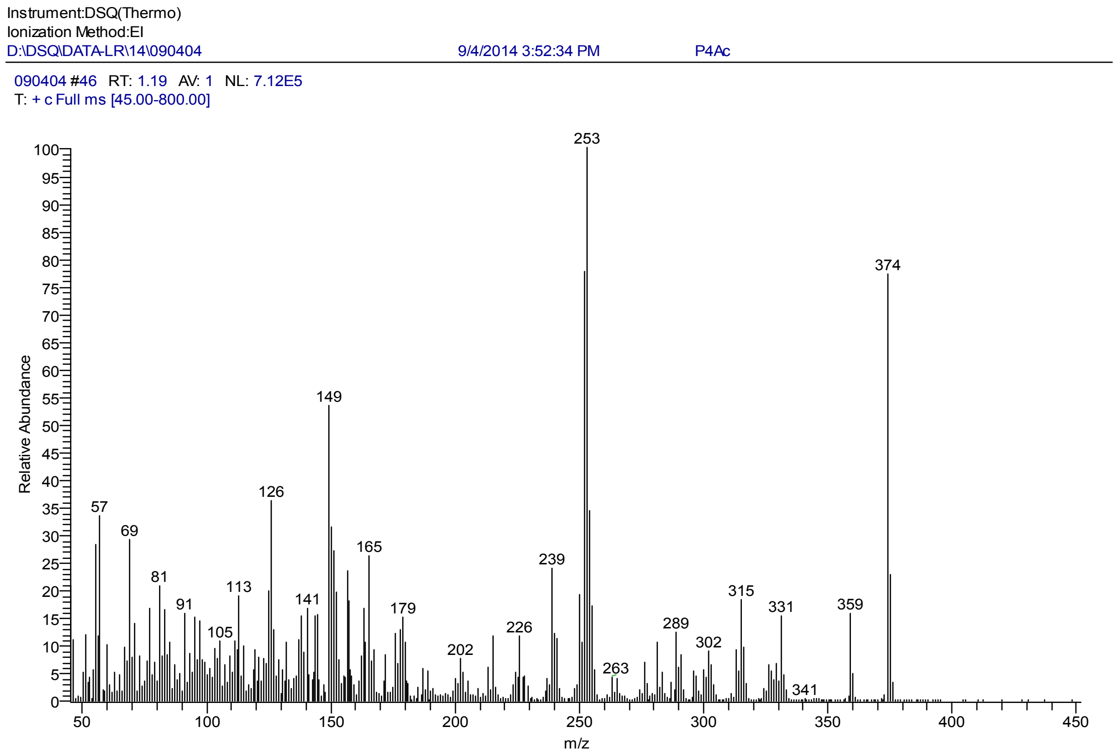

Fig. S14 EI-MS of *p*-P_4Ac

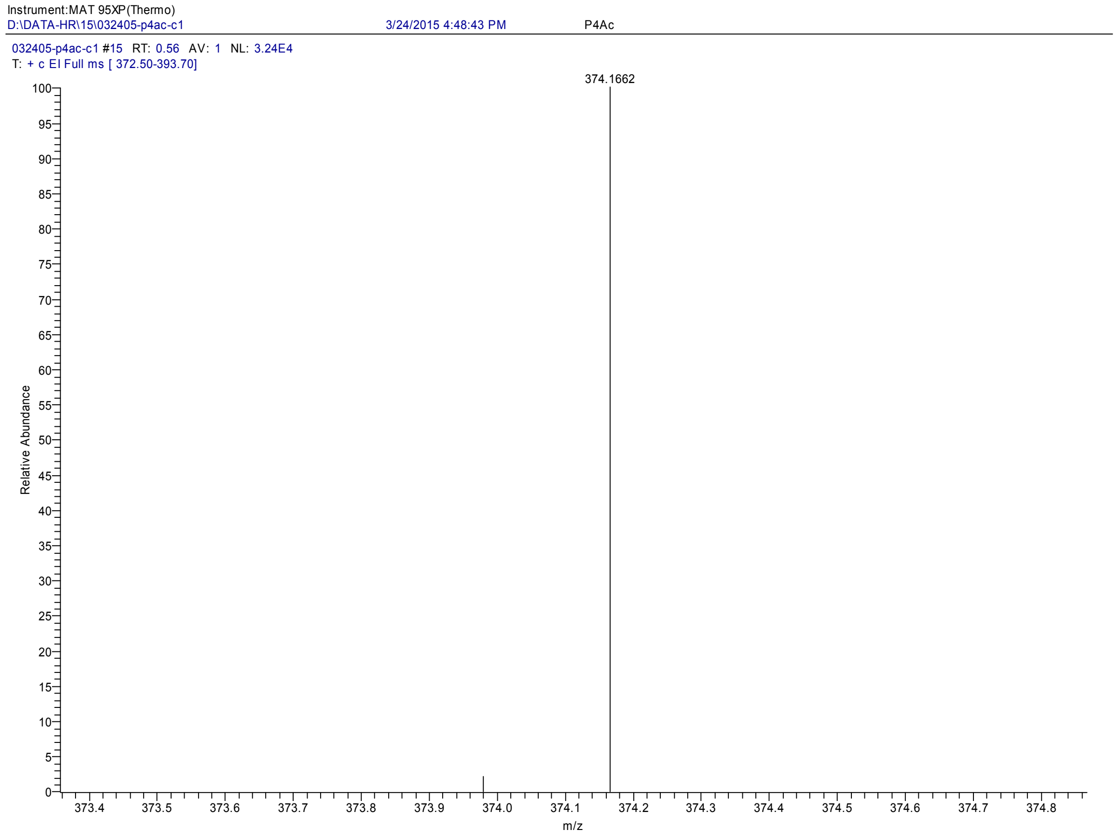

Fig. S15 HRMS of *p*-P_4Ac

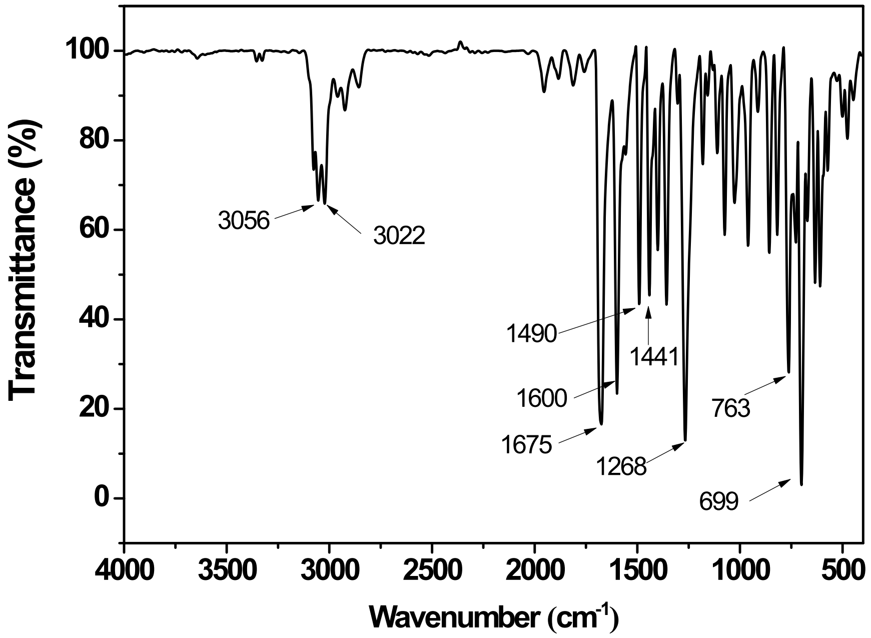

Fig. S16 FT-IR spectrum of *p*-P_4Ac

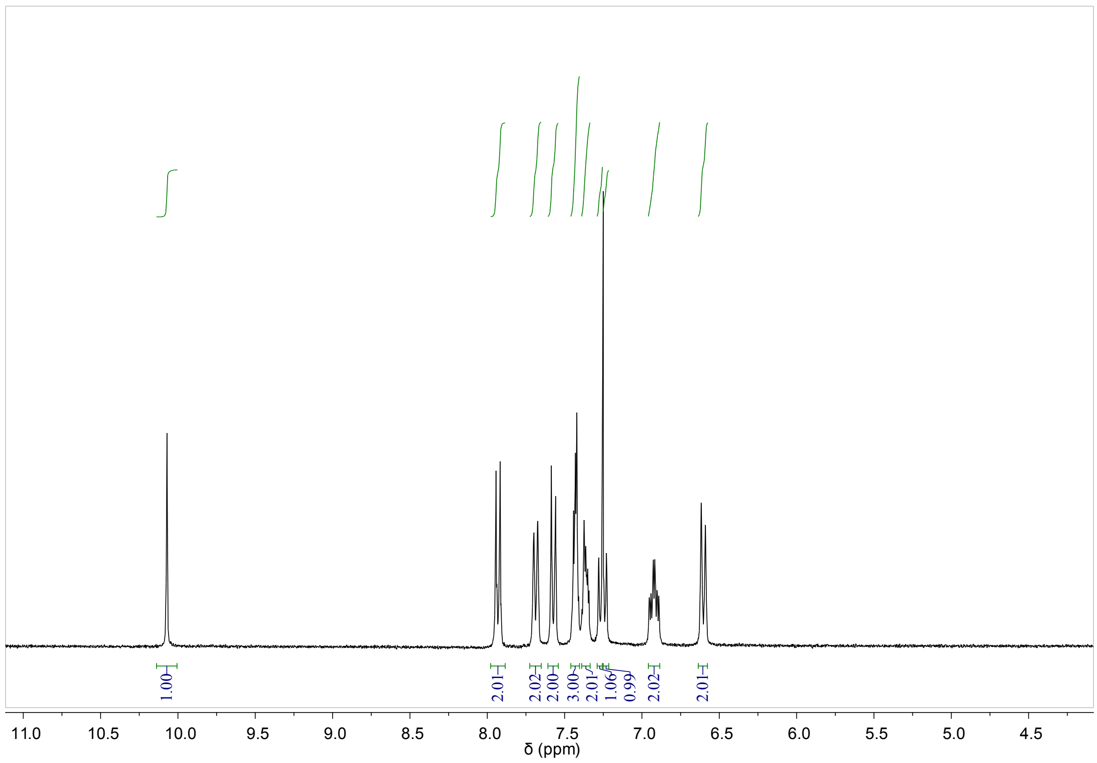

Fig. S17 ^{1}H NMR spectrum of *p*-FP$_2$A in CDCl$_3$

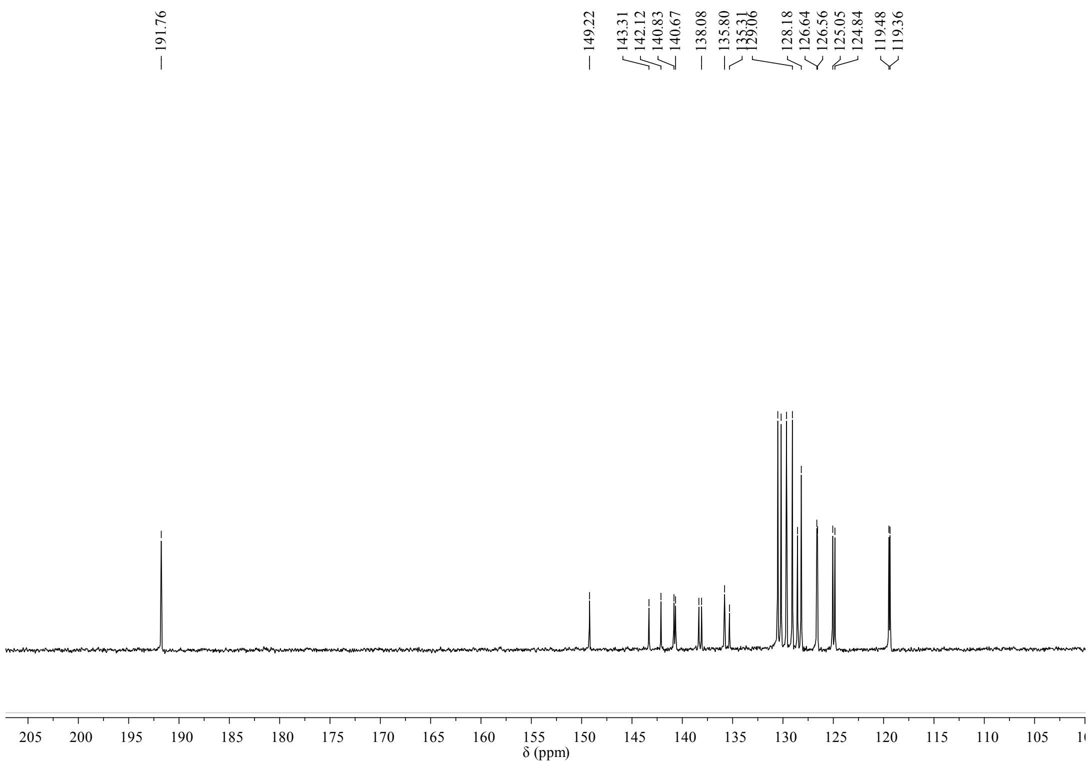

Fig. S18 ^{13}C NMR spectrum of *p*-FP$_2$A in CDCl$_3$

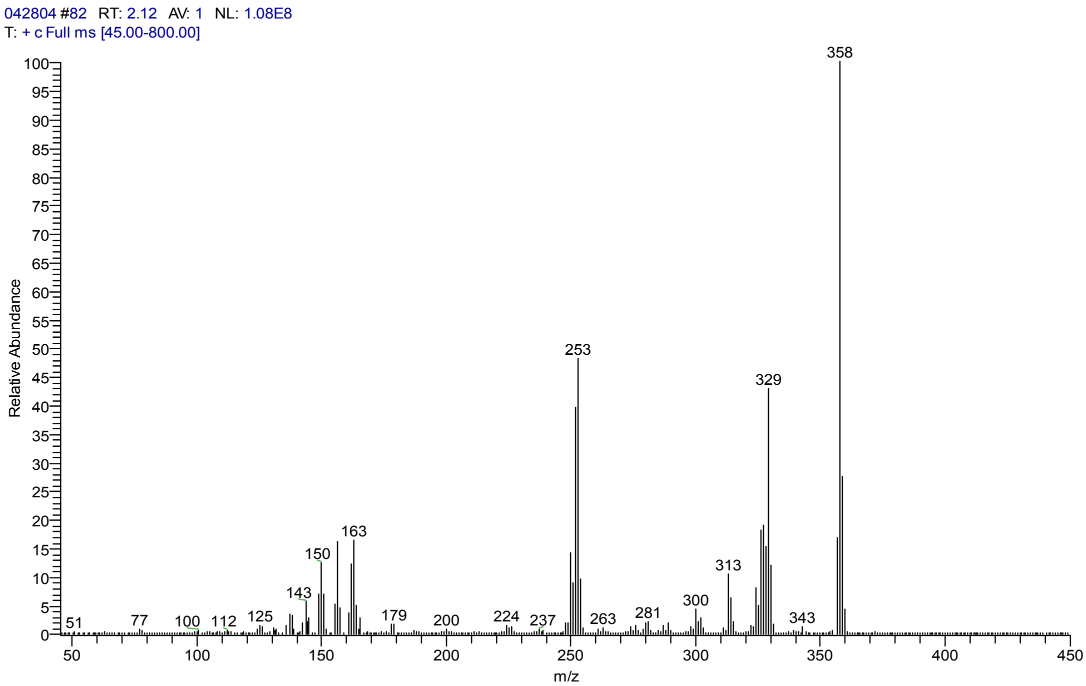

Fig. S19 EI-MS of *p*-FP_2A

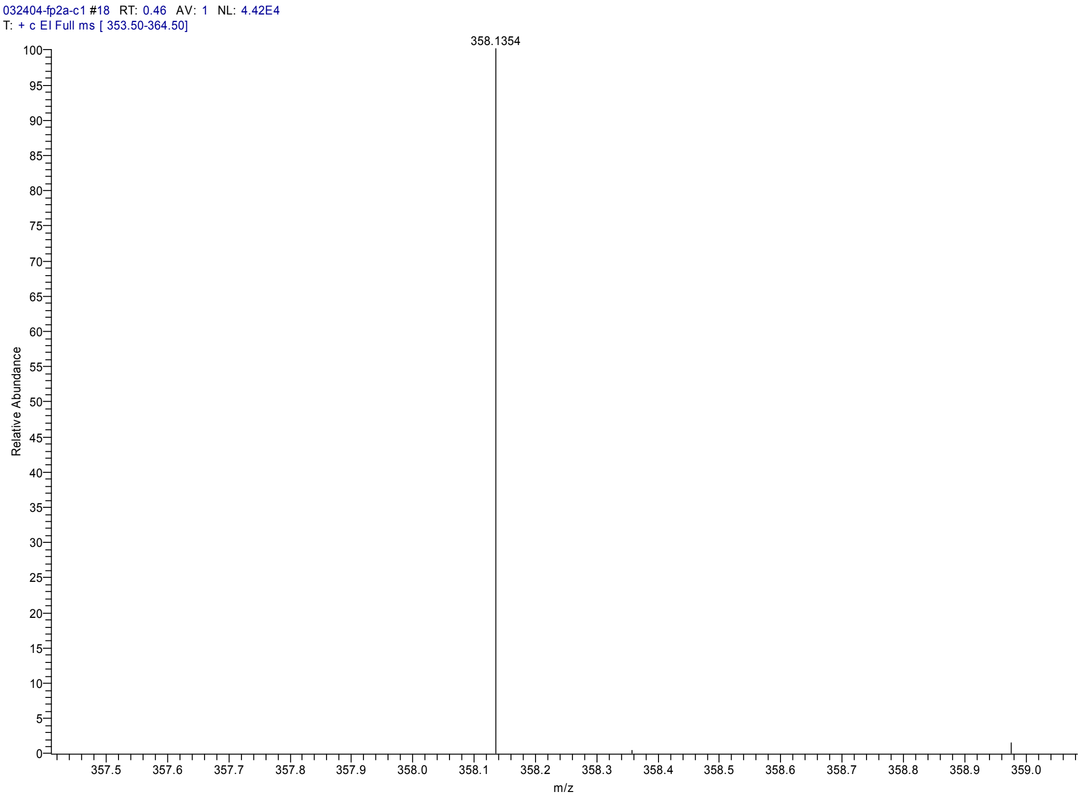

Fig. S20 HRMS of *p*-FP_2A

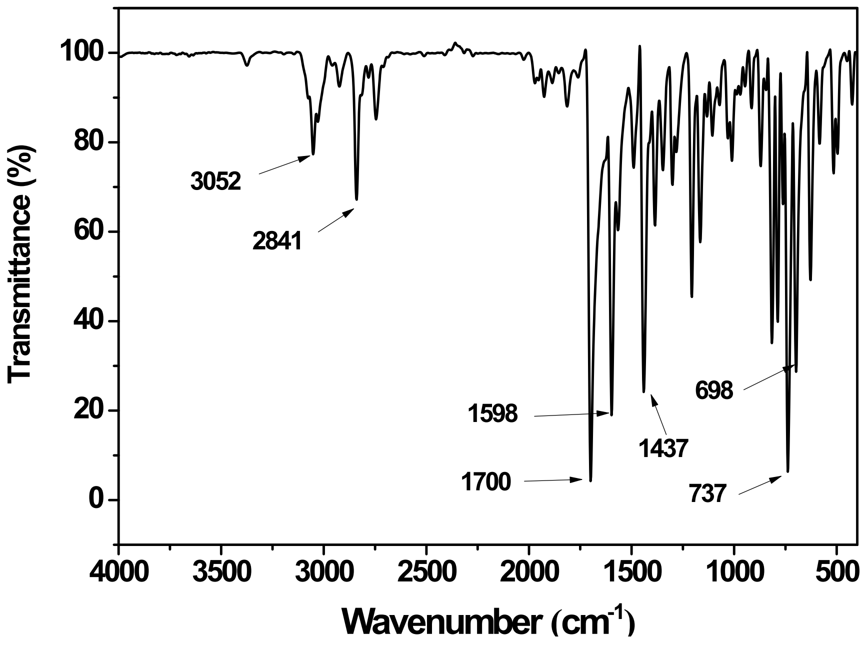

Fig. S21 FT-IR spectrum of *p*-FP_2A

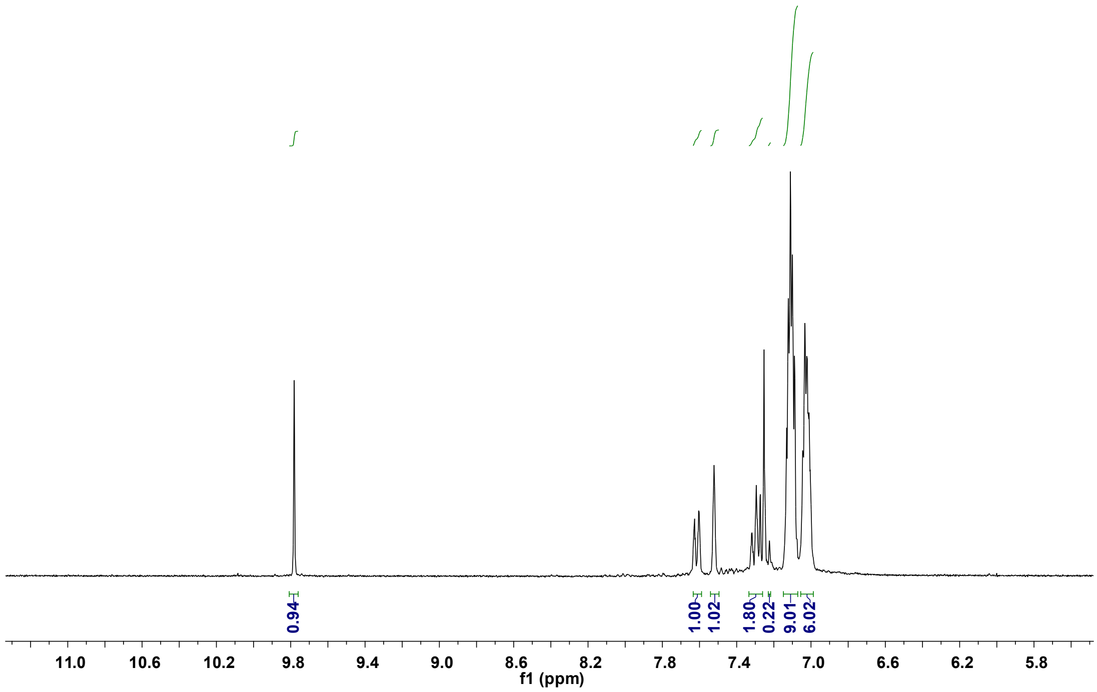

Fig. S22 ^1H NMR spectrum of *m*-P_4A in $CDCl_3$

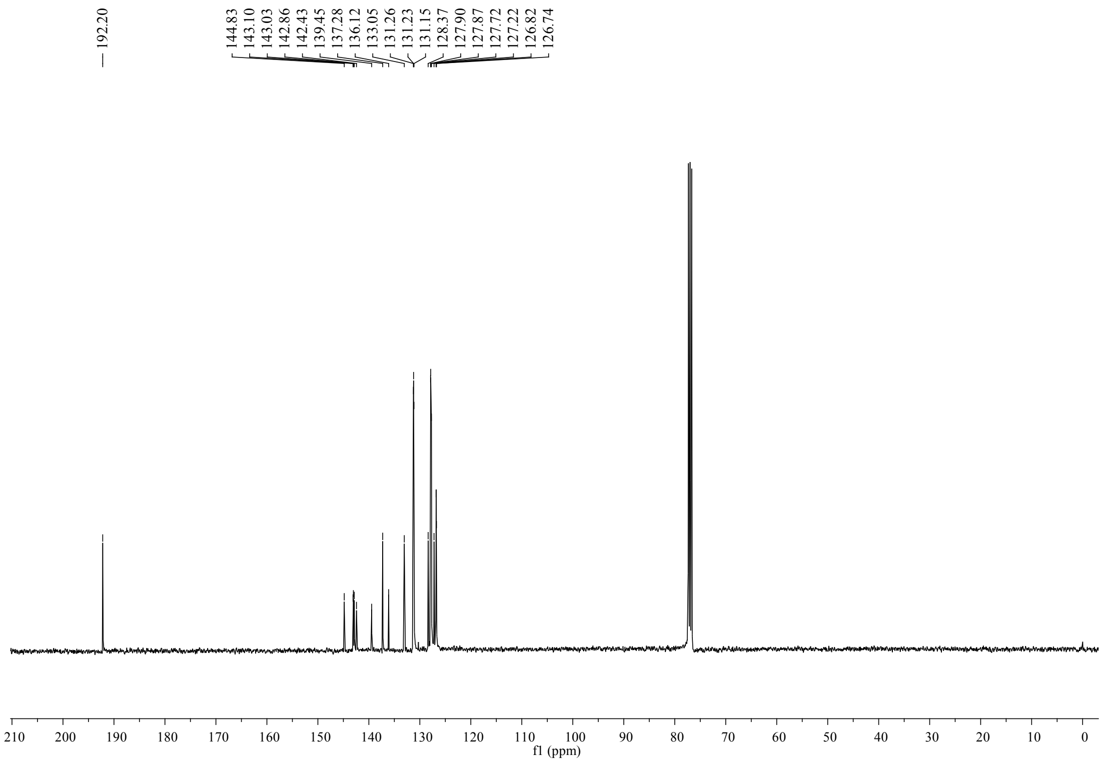

Fig. S23 ^{13}C NMR spectrum of *m*-P_4A in $CDCl_3$

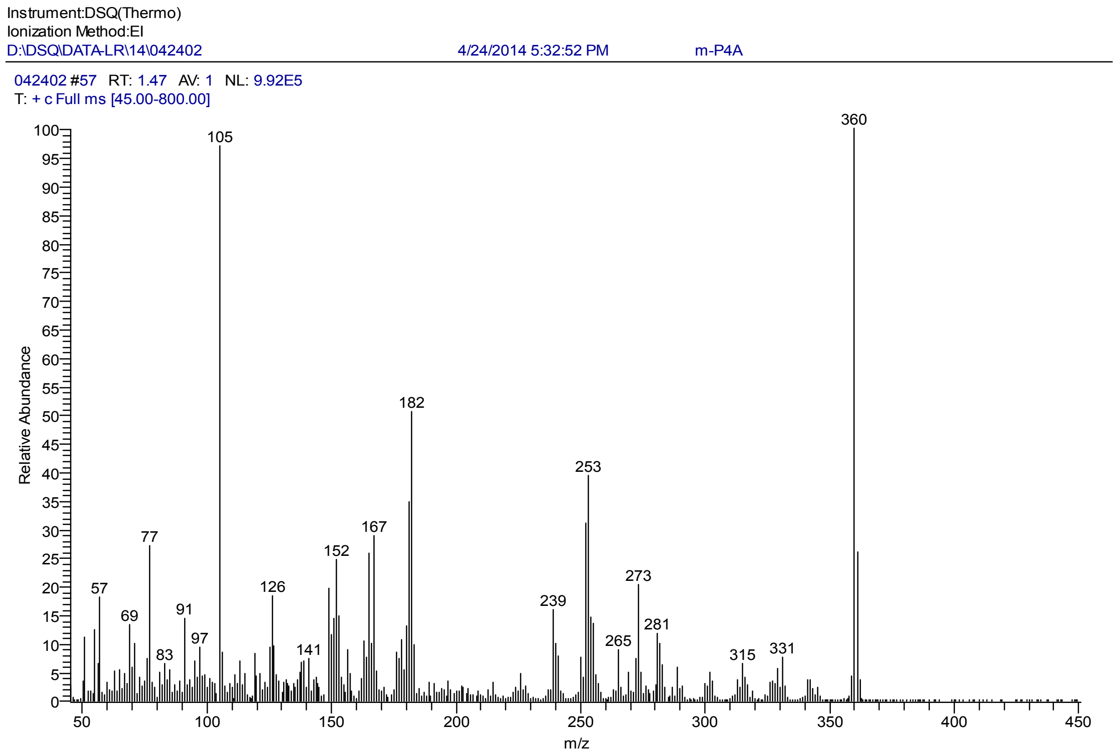

Fig. S24 EI-MS of *m*-P_4A

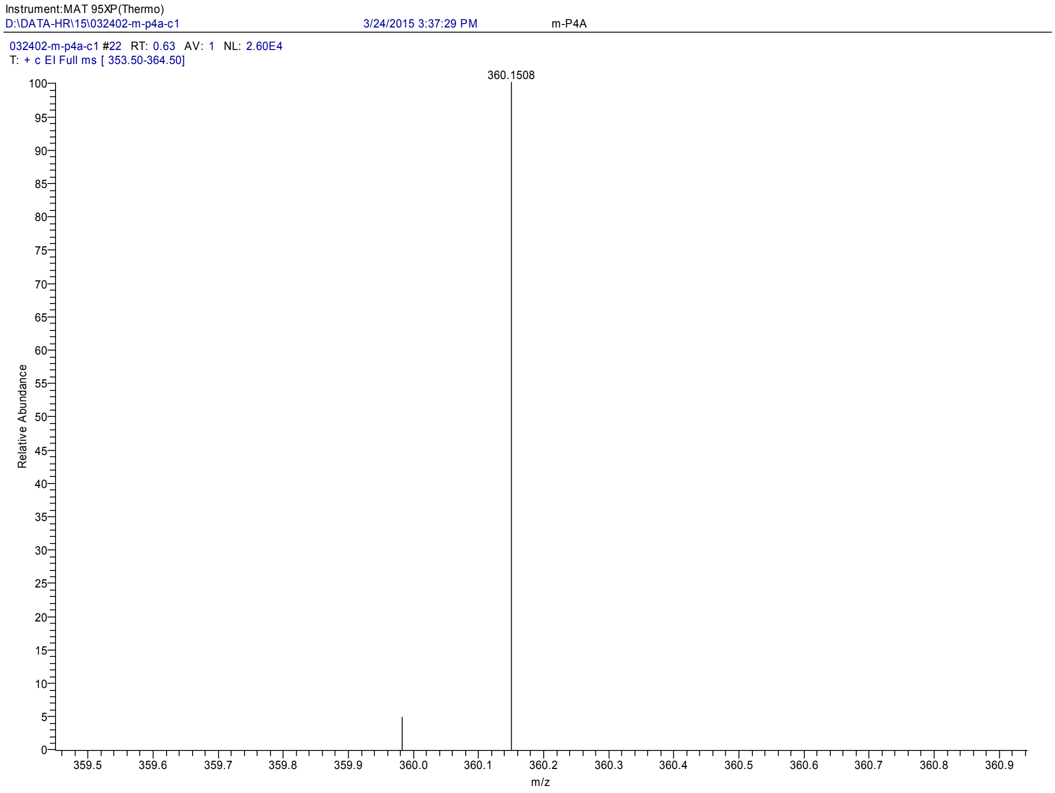

Fig. S25 HRMS of *m*-P_4A

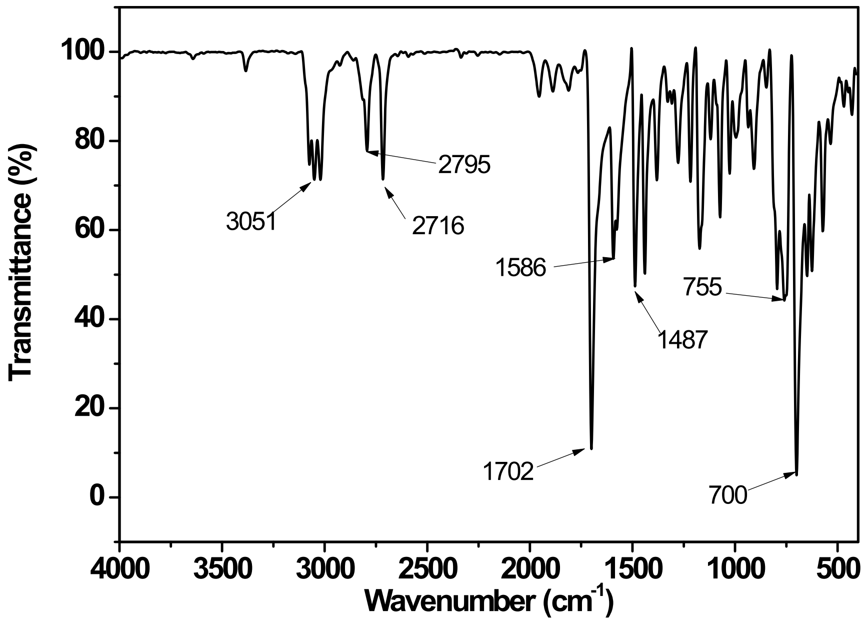

Fig. S26 FT-IR spectrum of *m*-P_4A

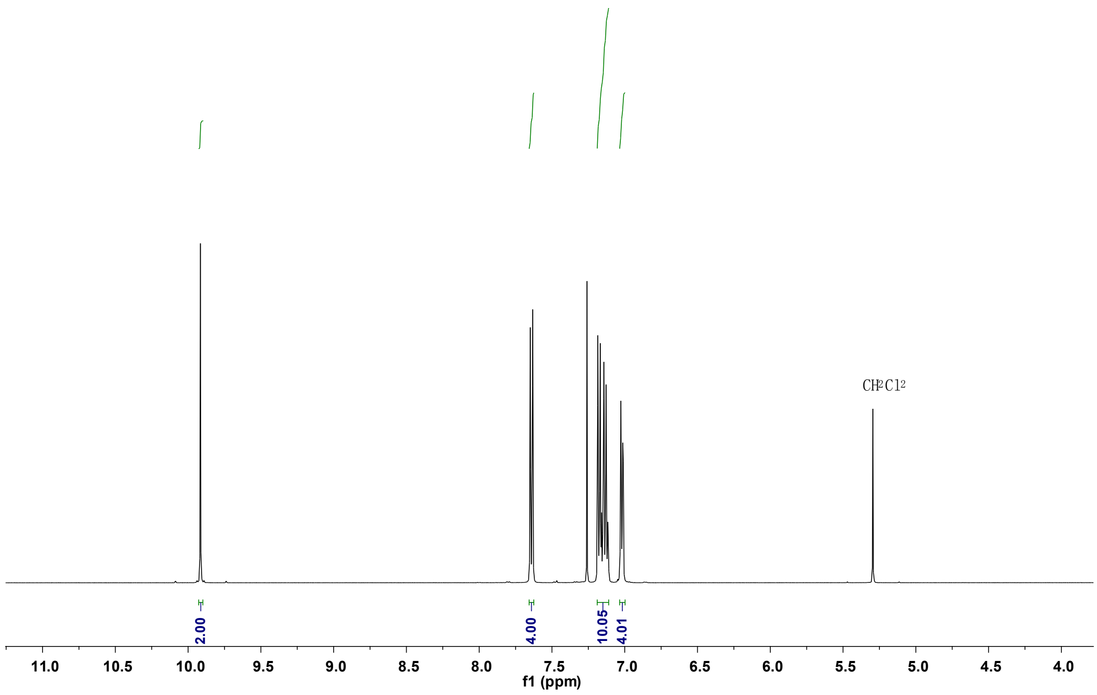

Fig. S27 ^{1}H NMR spectrum of *p*-P_4A_2 in $CDCl_3$

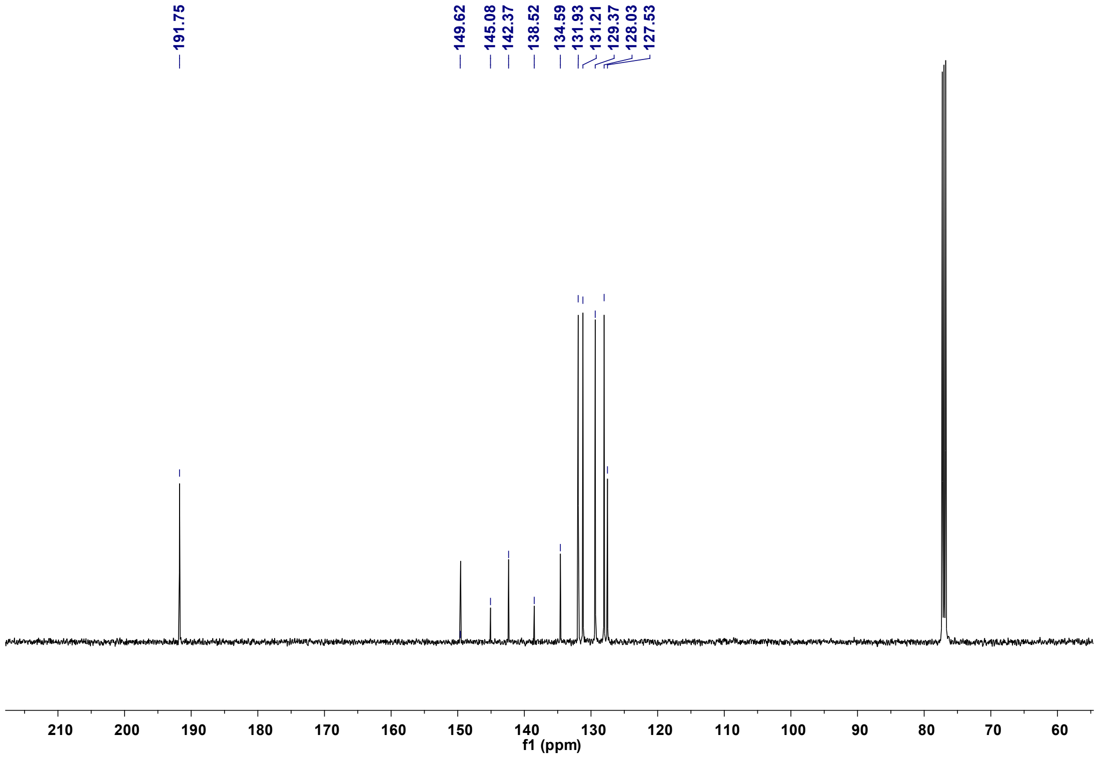

Fig. S28 ^{13}C NMR spectrum of *p*-P_4A_2 in $CDCl_3$

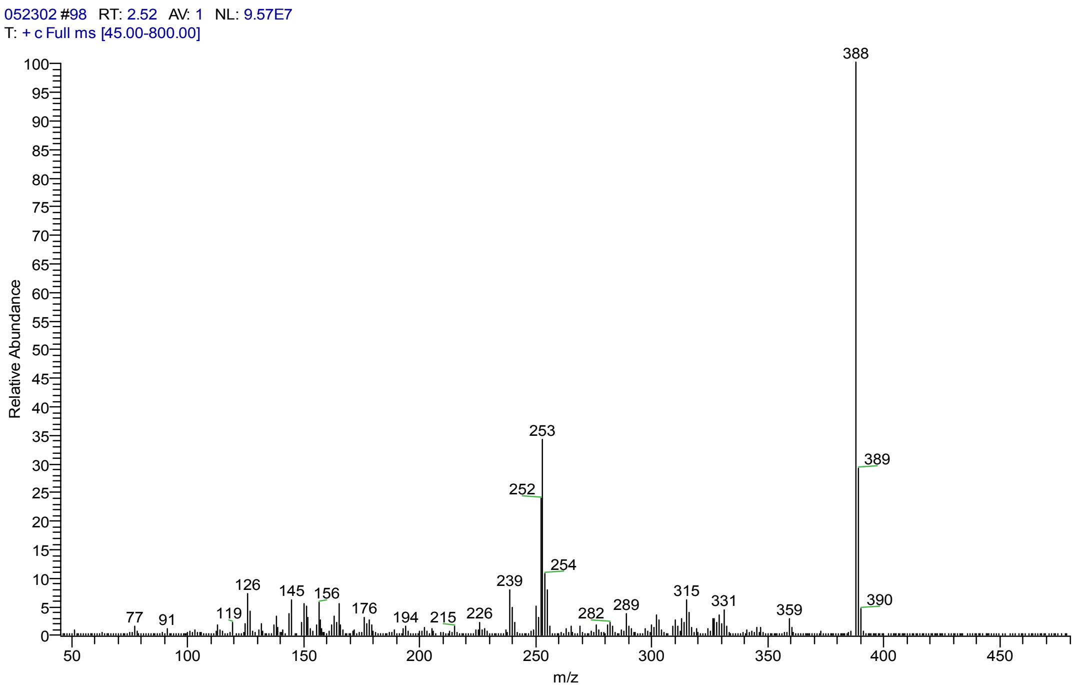

Fig. S29 EI-MS of p-P_4A_2

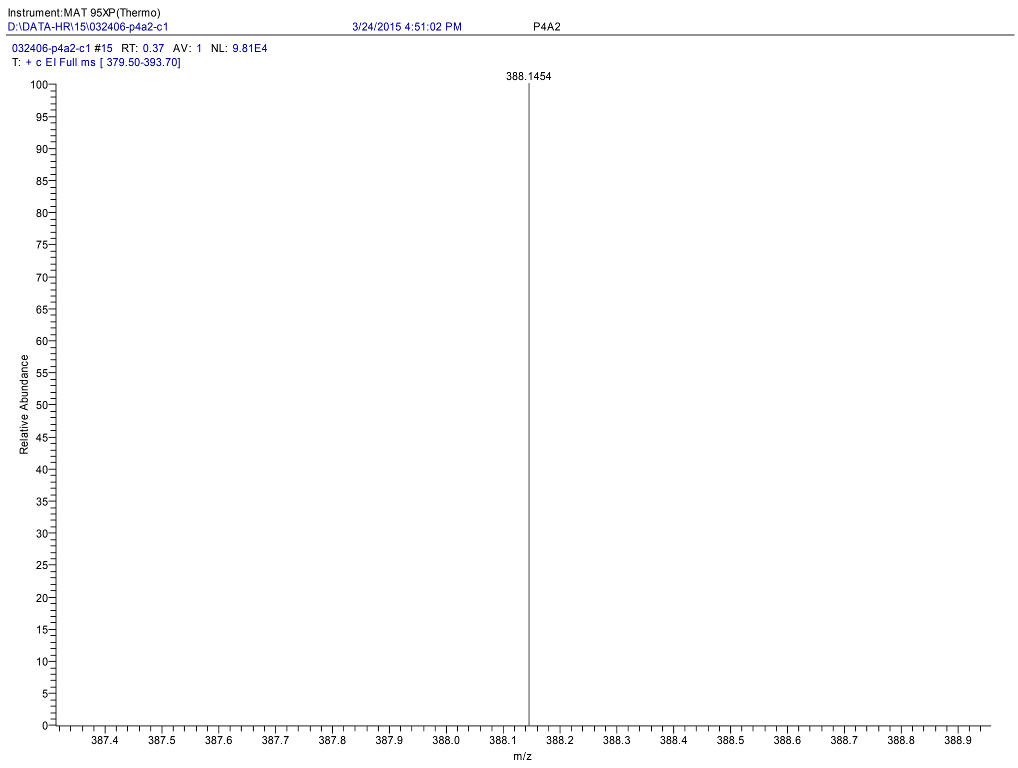

Fig. S30 HRMS of p-P_4A_2

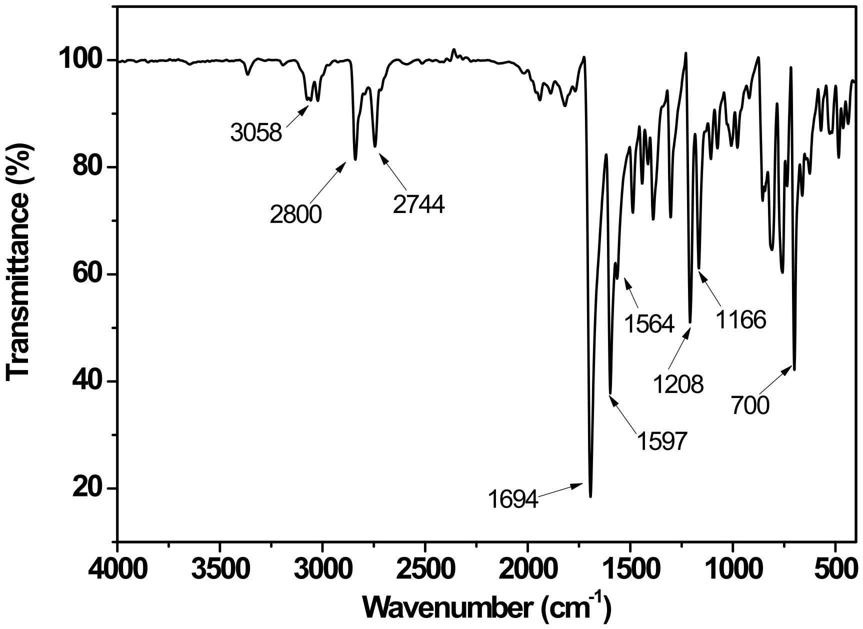

Fig. S31 FT-IR spectrum of *p*-P_4A_2
